# NAD^+^ metabolism: pathophysiologic mechanisms and therapeutic potential

**DOI:** 10.1038/s41392-020-00311-7

**Published:** 2020-10-07

**Authors:** Na Xie, Lu Zhang, Wei Gao, Canhua Huang, Peter Ernst Huber, Xiaobo Zhou, Changlong Li, Guobo Shen, Bingwen Zou

**Affiliations:** 1 State Key Laboratory of Biotherapy and Cancer Center, West China Hospital, and West China School of Basic Medical Sciences & Forensic Medicine, Sichuan University, and Collaborative Innovation Center for Biotherapy, Chengdu, 610041 China; 2 State Key Laboratory of Biotherapy and Cancer Center, West China Hospital, and Collaborative Innovation Center for Biotherapy, Chengdu, 610041 China; 3 School of Basic Medical Sciences, Chengdu University of Traditional Chinese Medicine, Chengdu, Sichuan 611137 China; 4 CCU Molecular and Radiation Oncology, German Cancer Research Center; Department of Radiation Oncology, Heidelberg University Hospital, Heidelberg, Germany; 5 First Department of Medicine, Medical Faculty Mannheim, University of Heidelberg, Theodor-Kutzer-Ufer 1-3, 68167 Mannheim, Germany; 6 West China School of Basic Medical Sciences & Forensic Medicine, West China Hospital, Sichuan University, Chengdu, 610041 China; 7 Department of Thoracic Oncology and Department of Radiation Oncology, Cancer Center, West China Hospital, Sichuan University, Chengdu, 610041 China

**Keywords:** Diseases, Molecular biology

## Abstract

Nicotinamide adenine dinucleotide (NAD^+^) and its metabolites function as critical regulators to maintain physiologic processes, enabling the plastic cells to adapt to environmental changes including nutrient perturbation, genotoxic factors, circadian disorder, infection, inflammation and xenobiotics. These effects are mainly achieved by the driving effect of NAD^+^ on metabolic pathways as enzyme cofactors transferring hydrogen in oxidation-reduction reactions. Besides, multiple NAD^+^-dependent enzymes are involved in physiology either by post-synthesis chemical modification of DNA, RNA and proteins, or releasing second messenger cyclic ADP-ribose (cADPR) and NAADP^+^. Prolonged disequilibrium of NAD^+^ metabolism disturbs the physiological functions, resulting in diseases including metabolic diseases, cancer, aging and neurodegeneration disorder. In this review, we summarize recent advances in our understanding of the molecular mechanisms of NAD^+^-regulated physiological responses to stresses, the contribution of NAD^+^ deficiency to various diseases via manipulating cellular communication networks and the potential new avenues for therapeutic intervention.

## Introduction

NAD^+^ was first described in 1906 as a component that could increase the fermentation rate in yeast.^1^ Years later, NAD^+^ was determined to play a vital role for hydrogen transfer in redox reaction.^2^ As an essential redox carrier, NAD^+^ receives hydride from metabolic processes including glycolysis, the TCA cycle, and fatty acid oxidation (FAO) to form NADH. NADH, therefore, serves as a central hydride donor to ATP synthesis through mitochondrial OXPHOS, along with the generation of ROS. Beyond its vital role as a coenzyme in energy metabolism, the important role of NAD^+^ has expanded to be a co-substrate for various enzymes including sirtuins, PARPs, CD157, CD73, CD38 and SARM1.^3–6^ Recently, it has been found that NAD^+^ serves as a nucleotide analog in DNA ligation and RNA capping.^7,8^ Therefore, the dynamic NAD^+^ and its metabolites levels, in response to diverse cellular stress and physiological stimuli, rewire biological processes via post-synthesis modification of fundamental biomolecules, including DNA, RNA and proteins.^9–13^ Through these activities, NAD^+^ impact energy metabolism, DNA repair, epigenetic modification, inflammation, circadian rhythm and stress resistance. NAD^+^ deficiency, however, contributes to a spectrum of diseases including metabolic diseases, cancer, aging and neurodegeneration disorders.

Here, we summarize recent advances in our understanding of the NAD^+^ homeostasis in response to growth conditions or environmental stimuli, highlighting the actions of NAD^+^ in coordinating metabolic reprogramming and maintaining cellular physiologic biology, which enables the plastic cells to adapt to environmental changes. Furthermore, we will discuss the NAD^+^ and its metabolites serving as an essential hub in both physiological and pathophysiological processes and explore the potential of NAD^+^ modulation in the clinical treatment of diseases.

## NAD^+^ homeostasis

NAD^+^, one of the most common metabolites in the human body, is in a homeostatic status of biosynthesis, consumption, recycling and degradation at both cellular and systemic levels.^14^

### NAD^+^ biosynthesis

#### De novo pathway

**Fig. 1:**
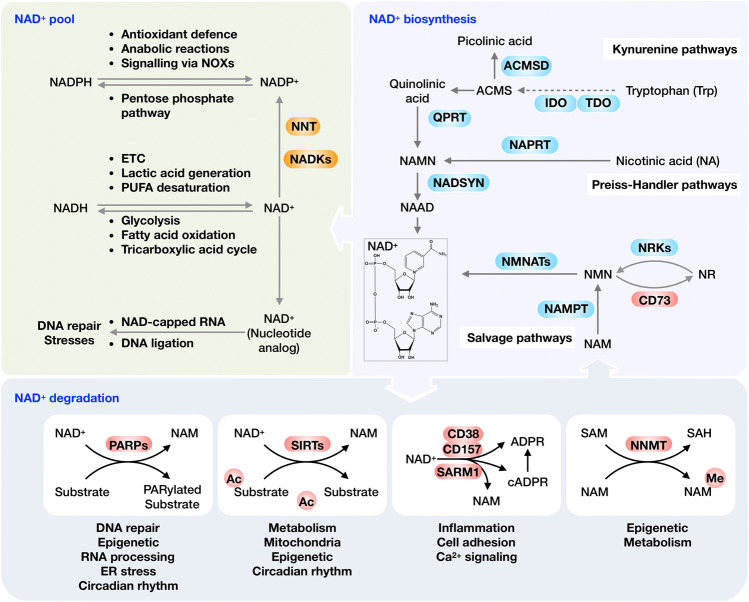
Overview of the NAD^+^ metabolism and its physiological function. Mammalian cells can synthesize NAD^+^ de novo from tryptophan by the kynurenine pathway or from NA by the Preiss‐Handler pathway, while most NAD^+^ is recycled via salvage pathways from nicotinamide (NAM), a by-production of NAD^+^-consuming reactions. NAD^+^ can be reduced into NADH in the metabolic processes, including glycolysis, fatty acid oxidation and the TCA cycle. NADH, in turn, drives the generation of ATP via OXPHOS, the production of lactic acid from pyruvate, and the desaturation of PUFAs. NADPH plays an essential role in antioxidant defense and regulates cellular signaling via NADPH oxidases (NOXs). Moreover, NAD^+^ is found to decorate various RNAs in different organisms as nucleotide analog and serves as an alternative adenylation donor for DNA ligation in NHEJ repair. NAD^+^ also acts as a co-substrate for a wide variety of enzymes, including PARPs, sirtuins, CD38/CD157 and SARM1, impacting metabolism, genomic stability, gene expression, inflammation, circadian rhythm and stress resistance. Using NAD^+^ as a co-substrate, both PARPs and sirtuins regulate their target molecules, generating NAM as a by-product. The CD38/CD157 and SARM1 also catalyze NAD^+^ to NAM, producing ADPR and cADPR. Abbreviations: IDOs, indoleamine 2,3-dioxygenase; QA, quinolinic acid; NAMN, nicotinate mononucleotide; QPRT, quinolinate phosphoribosyl-transferase; NAPRT, nicotinic acid phosphoribosyltransferase; NMNATs, nicotinamide mononucleotide adenylyl transferases; NADSYN, NAD synthase; NR, nicotinamide riboside; Trp, tryptophan; NADKs, NAD^+^ kinases; PARPs, poly (ADP-ribose) polymerases; NNT, nicotinamide nucleotide transhydrogenase; TDO, tryptophan 2,3-dioxygenase; SARM1, sterile alpha and TIR motif containing 1; NNMT, Nicotinamide N-methyltransferase; NMN, nicotinamide mononucleotide; PUFAs, polyunsaturated fatty acids; NAM, nicotinamide

Mammalian cells can generate NAD^+^ de novo from dietary tryptophan (Trp) by the kynurenine pathway (KP), which is initialized by either TDO or IDO. The intermediate ACMS can cyclize spontaneously to QA. However, ACMSD converts ACMS to picolinic acid, limiting the flux from tryptophan to NAD^+^.^15^ Another critical step catalyzes the conversion of QA to NAMN by QPRT, which commits the pathway to NAD^+^ biosynthesis.^16,17^ The Preiss-Handler pathway can convert dietary NA to NAMN by NAPRT.^18^ NAMN derived from both the tryptophan and NA is catalyzed by NMNATs to yield NAAD, which is then amidated to NAD^+^ by NAD synthase (NADSYN) using glutamine as nitrogen donor (Fig. 1).^19,20^

#### Salvage pathway

Rather than generated de novo, most NAD^+^ is recycled from NAM, NA, NR and NMN in the salvage pathway to maintain the cellular NAD^+^ levels.^21^ Among these precursors, NAM could be recycled from NAD^+^ consumption reactions, including both NAD^+^-dependent deacylation and ADP-ribosylation, into NMN by NAMPT, which catalyzes the rate-limiting reaction in the salvage pathway.^22^ The precursor NR is imported by ENTs and transformed to NMN by NRK1/2.^23^ Ultimately, NMN is adenylylated by NMNAT to yield NAD^+^^24,25^ (Fig. 1).

### NAD^+^ degradation

#### NAD^+^ consumption

As a co-substrate important to various postsynthesis modifications of fundamental macromolecules, NAD^+^ can be cleaved by NAD^+^-consuming enzymes including PARPs, sirtuins, CD38 and SARM1 to generate NAM and ADP-ribose (ADPR) (Fig. 1). The sirtuins are NAD^+^-dependent deacetylases that are distributed in the nucleus (e.g., SIRT1, SIRT6, and SIRT7), the cytoplasm (e.g., SIRT2) and mitochondria (e.g., SIRT3-5), respectively.^26^ Through mediating the post-translational modification dependent on NAD^+^, sirtuins modulate the adaptation to the altered cellular energetic status, especially the activation of oxidative metabolism and stress resistance in mitochondria in various physiological or pathological conditions.^26^ PARPs catalyze reversible ADP-ribosylation of macromolecular targets including proteins, DNA and RNA, utilizing NAD^+^ as a cofactor to provide monomer or polymers of ADP-ribose nucleotide.^27,28^ PARP members can be categorized into several groups, the poly-ADP-ribosyl transferases (e.g., PARP1, 2, and 5), the mono-ADP-ribosyl transferases (e.g., including PARP 3, 4, 6–8, and 10–16) and RBPs (e.g., PARP7, 10, and 12–14).^27,29^ PARPs-mediated ADP-ribosylation (ADPr) plays an essential role in cellular physiological processes in response to stimuli, particularly oxidative stress-induced DNA damage. Sustained PARP activation triggered by intense insults can cause NAD^+^ depletion and subsequent cell death.^30^ CD38 consumes NAD^+^ to make the calcium-releasing second messengers including ADPR (major product), 2^0^-deoxy-ADPR (2dADPR), NAADP and cADPR, contributing to age-related NAD^+^ decline.^31,32^ SARM1 is an important NAD^+^ consumer in neurons. The dimerization of TIR domain cleaves NAD^+^ into ADP-ribose, cADPR, and nicotinamide.^33–35^

NAD^+^-consuming enzymes seem to have a different Michaelis constant (*K*_m_) value for NAD^+^. The *K*_m_ of SIRT1 and SIRT3 ranges from 94 to 888 μM, which renders their activity tightly fluctuating with the dynamic physiological cellular NAD^+^ levels. Other sirtuins, including SIRT2, SIRT4, SIRT5 and SIRT6, have a *K*_m_ for NAD^+^ below the physiological range, implying that NAD^+^ might not necessarily be the rate-limiting of their activity.^5,36–46^ PARP-1, accounting for approximately 90% of the NAD^+^ used by the PARP family, has a lower *K*_m_ for NAD^+^ in the range of 20–97 μM.^47–49^ Of note, the CD38 and SARM1 display *K*_m_ for NAD^+^ in a markedly low micromolar range (15–25 μM).^50^ Based on their different *K*_m_ values, NAD^+^-consuming enzymes display various potential of reducing NAD^+^. Under normal homeostatic conditions, CD38 is expressed at low levels, whereas rising expression of CD38 with aging plays a vital role in age-associated NAD^+^ reduction.^51,52^ 78c, a highly potent and specific CD38 inhibitor, increases NAD^+^ levels, leading to activation of sirtuins and PARPs.^53^ Generally, the reported *K*_m_ of PARP1 and CD38 for NAD^+^ are lower than those of the sirtuins, suggesting that elevated activation of PARP1 or CD38 may limit endogenous SIRT activation by reducing NAD^+^ content. This notion is confirmed by the observation that PARP1 and CD38 inhibition effectively increases total NAD^+^ availability, leading to SIRT1 activation.^54^

#### NAD^+^ methylation

Excess NAM that is not recycled is metabolized through two enzymatic systems and eventually excreted from the body.^55^ The first system methylates the NAM into MNAM by NNMT, which utilizes the SAM as methyl donor.^56^ The MNAM together with their oxidized compounds, 4py and 2py, are eventually eliminated in the urine.^57^ This methylation system is quantitatively by far the predominant NAM scavenging pathway under most conditions. While an acute pharmacological dose of NAM can be converted by CYP2E1 to nicotinamide N-oxide, which is then excreted to the urine.^55,58,59^ Therefore, NNMT and CYP2E1 divert NAM from recycling to NAD^+^, restraining NAM accumulation and inhibition of NAD^+^-dependent signaling.^60^ The Km of the human NNMT enzyme for NAM (approximately 430 μM) is much higher than the affinity of NAMPT for NAM (<1 μM), suggesting an unsaturated NNMT under normal conditions. Increasing dietary NAM intake can lead to a proportional increase in NAM methylation.^61,62^ Further, elevated NNMT expression or increased MNAM levels in the liver can stabilize SIRT 1, which in turn promotes glucose and cholesterol metabolism (Fig. 1).^63^

### Subcellular distribution of NAD^+^

#### NAD^+^/NADH

Both the oxidized NAD(P)^+^ and the reduced NAD(P)H have redox and signaling functions with an uneven subcellular distribution. As listed in Box 1, a portfolio of approaches is developed to map the total quantification or cellular concentrations of the four NAD^+^ coenzymes. Semisynthetic fluorescent biosensor-based analysis of U2OS cells exhibits around 70 μM for cytoplasmic NAD^+^, ~110 μM for nuclear NAD^+^ and ~90 μM for mitochondrial NAD^+^, respectively. Meanwhile, the concentration of free cytosolic NAD^+^ detected in cell lines including U2OS, HEK293T, NIH/3T3 and HeLa is ranging from 40 to 70 µM.^64–67^ The similar depletion rate of free NAD^+^ in the cytoplasm and nucleus supports the notion regarding a probable exchange of NAD^+^ between these compartments, while a slower rate of free NAD^+^ depletion in mitochondrial suggests that the mitochondrial NAD^+^ pool is segregated from the cytosolic and nuclear pools.^65,68^ In agreement with these reports, mounting evidence implies that the distinct fluctuation of NAD^+^ in mitochondria may be attributed to the membrane impermeability of NAD(H).^69,70^ Controversially, isotope-tracer method analysis shows that mammalian mitochondria are capable of taking up intact NAD^+^ as well as its precursors, such as NMN and NR.^37,71–73^ Although NAD^+^ transporter has been identified in bacteria, yeast and plants, no mammalian homolog has been discovered so far to validate the import of NAD^+^ into mitochondria.^74–78^

**Fig. 2:**
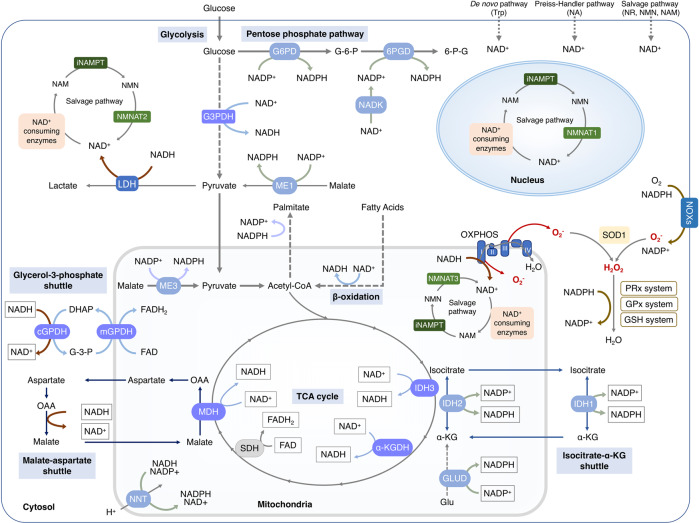
Subcellular equilibrium of NAD^+^. The NAD^+^ homeostasis is maintained by the biosynthesis, consumption and recycling in differentiate subcellular compartments including the cytosol, the nucleus and the mitochondria. NAD^+^ precursors including Trp, NA, NR, NMN and NAM are metabolized into NAD^+^ via Preiss-Handler pathway, de novo pathway and salvage pathway, respectively. NAD^+^ can receive hydride to yield the reduced form NADH in the metabolic processes including glycolysis, FAO, and the TCA cycle. NADH provides an electron pair to drive the mitochondrial OXPHOS for the generation of ATP and the conversion of lactic acid to pyruvate. The cytosolic and mitochondrial NADH is exchanged through the malate-aspartate shuttle and glycerol-3-phosphate shuttle, while the cytosolic and mitochondrial NADPH is exchanged by the isocitrate-a-KG shuttle. NAD^+^ can also be phosphorylated into NADP^+^ by NAD^+^ kinases including nicotinamide nucleotide transhydrogenase (NNT) and NAD kinases (NADKs). Cytosolic NADP^+^ is reduced into NADPH by G6PD and 6PGD in the pentose phosphate pathway, and by ME1 in the conversion of malate to pyruvate. Mitochondrial NADPH is produced by IDH2, GLUD, NNT and ME3. The NADPH is required for the activation of NOXs and the synthesis of palmitate. Abbreviation: α-KGDH, alpha-ketoglutarate dehydrogenase; GLUD, glutamate dehydrogenase; NNT, nicotinamide nucleotide transhydrogenase; G3PDH, glyceraldehyde 3-phosphate dehydrogenase; 6PGD, 6-phosphogluconate dehydrogenase; G6PD, glucose-6-phosphate dehydrogenase; GPx, glutathione peroxidases; IDH1/2, isocitrate dehydrogenase 1 and 2; MDH, malate dehydrogenase; ME1/3, malic enzyme; NADK, NAD^+^ kinase; NOXs, NADPH oxidases; OXPHOS, oxidative phosphorylation; PPP, pentose phosphate pathway; PRx, peroxiredoxin; SDH, succinate dehydrogenase; SOD1-3, superoxide dismutase type 1-3; TCA cycle, tricarboxylic acid cycle; GSH, Glutathione; LDH, Lactate dehydrogenase

The NAD^+^ pool in each cellular compartment can also be maintained independently via recycling the NAD^+^ from NAM, dependent on various forms of NMNATs, e.g., the nucleic NMNAT1, cytosolic NMNAT2 and mitochondrial NMNAT3.^79^ Nevertheless, the independent mechanism for maintaining NAD^+^ through salvaging NMN in mitochondria is challenged by the controversy around the presence or absence of NAMPT and NMNAT3 in mitochondria.^80,81^ The electrons of NADH, rather than NADH itself, generated from glycolysis in the cytoplasm could be transferred across the mitochondrial membrane through the NADH shuttle systems.^66,82^ The glycerol-3-phosphate (G-3-P) shuttle and malate-aspartate shuttle transfer the electrons from cytosolic NADH to mitochondrial FADH2 or NADH, respectively. Then, the electrons are finally transferred to the ETC^83–90^ (Fig. 2).

#### NADP^*+*^*/NADPH*

Approximately 10% of cellular NAD^+^ may be phosphorylated by NAD^+^ kinases into NADP^+^, which can be dephosphorylated to NAD^+^ by NADP^+^ phosphatases.^91,92^ Cytosolic NADPH, the reduced form of NADP^+^, is mainly generated in the pentose phosphate pathway (PPP) involving G6PD and 6PGD. The mitochondrial NADPH can be produced by ME3 that converts pyruvate to malate and by IDH2 that catalyzes isocitrate to α-ketoglutarate.^93,94^ Additionally, NADP^+^ can also receive the electrons from NADH to form NADPH by NNT that locates in the mitochondrial inner membrane.^95^ These distinct synthesis pathways might contribute to the differential subcellular NADPH/NADP^+^ ratio, such as a significantly higher ratio in mitochondria (~170) than that of the cytosol and the nucleus (40~50) in U2OS cells.^64^ NADPH is required for both the reductive biosynthetic reactions of cholesterol and palmitate and the oxidative reactions catalyzed by NADPH oxidases (NOXs), nitric oxide synthases (NOS), cytochrome P-450, and so on.^95,96^ Most importantly, NADPH provides the primary reducing power for the thioredoxin (Trx) and glutathione (GSH) systems to eliminate ROS (Fig. 2). In line with that, the free NADPH/NADP^+^ ratio that indicates the reduction potential is normally sustained at a high level inside cells and is significantly reduced following pro-oxidant agents or H_2_O_2_ exposure.^64,96,97^ Given its essential role in metabolism and antioxidant defense, NADPH/NADP^+^ ratio in cancer cells exhibiting high metabolic rate and ROS contents (50-70) is much higher than that in the embryonic kidney immortalized cell line HEK293 (~20).^64^ Albeit further research is needed, quantification of NADPH/NADP^+^ ratios provides an effort to map metabolic and redox state of different cell types and organelles.

BOX 1 NAD(P)^+^/NAD(P)H detection assays **Biochemical analysis**

The biochemical analysis uses enzymatic cycling assays, capillary electrophoresis (CE), or high‐performance liquid chromatography (HPLC) coupled with mass spectrometry (LC/MS/MS), to detect the NAD^+^ and NADH contents and the NAD^+^/NADH ratio.^80,654–658^

The enzymatic cycling assays is based on a multi-step NAD^+^/NADH enzyme cycling reactions that convert WST-1 to WST-1 formazan, which can be easily detected at OD 450 nm. This assay is recommended to evaluate the effect of activators and inhibitors on NAMPT activity using purified protein and to check for contamination and interference for NAD^+^ present in the sample, such as immunoprecipitated cell lysates. However, this approach measures the total quantity of cellular NAD^+^ or NADH, regardless of the free and protein‐bound forms or the differentiated subcellular compartmentation. Additionally, the requirement of tissue biopsy and extraction renders the enzymatic cycling assays incompatible with longitudinal studies in intact organs.^654–656^

Based on the enzymatic cycling reaction, a CE approach is established to measure NAD^+^ and NADH in a single cell in a single run with a capillary electrophoresis laser-induced fluorescence (CE-LIF) setup. This method shows good reproducibility and specificity with a detection capability as low as 0.2 amol of NAD^+^ and 1 amol of NADH.^659,660^

HPLC coupled MS can simultaneously analyze the four coenzymes including NAD^+^, NADH, NADP^+^, and NADPH, and the related metabolites. This approach provides accurate, sensitive, reliable, rapid, and reproducible results, which enables us to map various pathophysiological alterations in NAD^+^ metabolism.^661–665^

**Autofluorescence approach**

The autofluorescence approach is a less invasive optical approach. Under ultraviolet excitation, NADH/NADPH exhibits identical autofluorescence signals, whereas the related oxidized forms NAD^+^/NADP^+^ are not fluorescent. The autofluorescence intensity has often been microscopic determined as the quantification of NAD(P)H. Additionally, fluorescence lifetime imaging microscopy (FLIM) is capable of differentiating the quantitative of free and protein‐bound NAD(P)H independent on intensity, interpreting as an indirect readout of cellular metabolism. Collectively, this method based on intensity and decay times of the autofluorescence allows the analysis of cellular redox state and metabolism in cells and tissues. However, the application of this marker-free approach is limited by its signal ambiguity, limited penetration and trouble in monitoring the autofluorescence signal from deep tissue or organs.^654,666^

**Genetically encoded fluorescent redox sensors**

The highly responsive, genetically encoded fluorescent sensors, including Frex, LigA-cpVenus, SoNar, Peredox, RexYFP for NAD^+^/NADH, iNap1-4 and Apollo-NADP^+^ for NADP^+^/NADPH, can image and monitor NAD(P)^+^/NAD(P)H redox state in living cells and in vivo. Advantages of the fluorescent redox sensors are able to determine subtle perturbations of the cellular energy metabolism in real-time. Meanwhile, it can be adapted to high-throughput chemical screening of potential compounds targeting cellular metabolism in a variety of analytical platforms, including microplate readers, flow cytometry, fluorescence microscopy and high-content imaging systems.^97,654,667–674^

^**31**^**P-magnetic resonance spectroscopy (**^**31**^**P-MRS) methods**

^31^P-MRS based NAD^+^ assay is a noninvasive method that could quantitatively measure intracellular NAD^+^ contents and redox state in animal and human tissues, such as brains. It provides new approach to investigate intracellular NAD^+^ redox state and metabolism in the human tissues with the potential for translation to human application.^654,675–678^

**Isotope-tracer methods**

Isotope labeling metabolites, including [2,4,5,6-^2^H] NAM, [U-^13^C] Trp, [U-^13^C] NA, and NR (nicotinamide 7-^13^C, ribose 2-^2^H), can be intravenous infused into mice or added into the media of cell culture. The labeled metabolites in cells, serum and tissues are analyzed by LC-MS. Isotope-tracer methods are applied in quantitative analysis of NAD^+^ synthesis-breakdown fluxes, including NAD^+^ synthesis and consumption fluxes in cell lines, as well as NAD^+^ fluxes in vivo.^91^

### NAD^+^ homeostasis at the systemic level

The NAD^+^ and its metabolites systemically flux and exchange across tissues, with a tissue-specific distribution of NAD^+^ biosynthetic enzymes and a tissue-specific preference for specific NAD^+^ precursors. It is reported that the de novo biosynthesis of NAD^+^ from tryptophan mainly occur in the liver and, to a lesser extent, kidney, which is attributed to the exclusively expressed enzymes involved in de novo NAD^+^ synthesis in these tissues. Therefore, the concentration of tryptophan in the diet affects the liver NAD^+^ levels. Tryptophan also compensates the NAD^+^ biosynthesis when the salvage pathway is blocked.^35,69,91^ NAM is the main NAD^+^ source in both cell lines and most murine tissues. The circulating NAM, 95% of which is released by the liver, is the main NAD^+^ source for the rest of the body. The NAM uptake preference differs dramatically, ranging from the highest 70 μM in spleen and small intestine to the lowest 2–9 μM in the white adipose and skeletal muscle. Besides tryptophan and NAM, NA is the third NAD^+^ precursor with concentrations >0.1 mM in mammalian plasma, which can only be used by spleen, small intestine, pancreas, kidney and liver.^91^ Accordingly, these tissues have been observed with a remarkable expression of NAPRT that guides NA into NAD^+^ biosynthesis.

Additionally, NMN and NR are capable of efficiently elevating tissue NAD^+^ concentrations. Given that NMN itself is a non-cell penetrating biosynthetic intermediates, NMN or its metabolites may be actively transported across the membrane. The solute carrier family 12 member 8 (Slc12a8) has been reported as a specific NMN transporter that is responsible for the uptake of NMN and maintenance of NAD^+^ level in the murine small intestine.^98,99^ However, it has been reported that the dephosphorylation of NMN into NR by extracellular 5’-nucleotidases is required for the uptake and utilization of NMN in cellular NAD^+^ synthesis.^98^ Similarly, the modification of the phosphate group in NMN allows its transportation to activate SARM1 in cells.^100^ Therefore, whether NMN is directly transported by Slc12a8 remains unclear, which needs further investigation. NR, which can cross the cell membrane through a dypiridamole-inhibitable nucleoside transporter, is preferentially used by muscle to synthesize NAD^+^.^98^ Accordingly, the NR-using enzyme, NRK2, is usually specifically expressed in the skeletal muscle.

Beyond the systemic regulation of NAD^+^ homeostasis across various tissues, it has recently been described that bacteria contribute to mammalian host NAD^+^ biosynthesis, especially following oral intake of the amidated precursors. The oral NAM and NR can be deamidated by gut microbiota into NA, NAR, NAAD and NAMN in the small intestine and the colon. These deamidated NAD^+^ metabolites are circulated to the liver and kidney, significantly contributing to the bulk of NAD^+^ biosynthesis.^101^

Despite major advances in the acknowledgment of tissue-specific NAD^+^ homeostasis, further work will be needed to fully characterize the subcellular and systemic modulation of NAD^+^ metabolism, which can improve the preventive and therapeutic strategies based on maintaining healthy NAD^+^ homeostasis.

## NAD^+^ metabolism in physiological function

Serving as crucial co-enzymes for redox reactions and co-substrates for NAD^+^-dependent enzymes, NAD^+^ and its metabolites function as a regulatory hub controlling a broad range of physiological processes, including redox homeostasis, genomic stability, gene expression, RNA processing, energy metabolism, immunity and inflammation, and circadian clock.

### NAD^+^ metabolism maintains the redox homeostasis

**Fig. 3:**
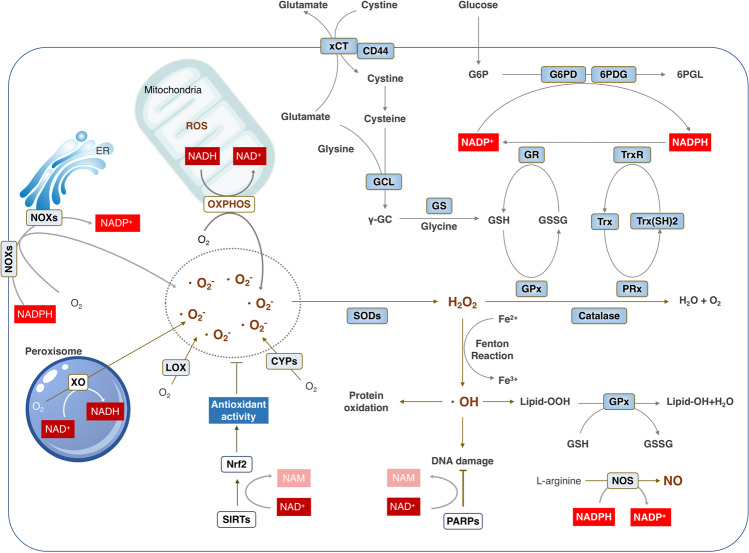
NAD^+^ metabolism controls the redox homeostasis. ROS could be produced from either metabolic reaction in mitochondria, such as OXPHOS, or from a range of cytosolic enzymes, including NOXs, XO, LOX, CYPs, all of which need the NADH/NADPH serving as the electron donor. To maintain the redox homeostasis, both enzymatic and non-enzymatic antioxidant system components exhibit their effects in coordination with each other to contract with the ROS. GSH, the most abundant of non-enzymatic antioxidants, is synthesized from glutamate, cysteine and glycine catalyzed by two consecutive cytosolic enzymes, GCL and GS. Importantly, NADPH serves as the reductive power for ROS-detoxifying enzymes including glutathione reductases (GR) and thioredoxin reductases (TrxR) to maintain the reduced forms of GSH and Trx (SH)_2_ in response to ROS produced from mitochondria or NOXs. Abbreviations: 6PGD, 6-phosphogluconate dehydrogenase; CYPs, Cytochromes P450; G6PD, glucose-6-phosphate dehydrogenase; GCL; GR, glutathione reductases; GS; LOX; NAD, nicotinamide adenine dinucleotide; NOXs, NADPH oxidases; NADPH, nicotinamide adenine dinucleotide phosphate; OXPHOS, oxidative phosphorylation; PRx, peroxiredoxin; GPx, glutathione peroxidases; SOD1/2, superoxide dismutase 1 and 2; Trx, thioredoxin; TrxR, thioredoxin reductases; XO, xanthine oxidase

Cells continuously generate oxidants and produce antioxidants. An imbalance between the oxidant formation and the antioxidant capacity in favor of the former causes oxidative stress.^102^ Maintenance of a physiological (low-level) oxidative stress, also denoted as oxidative eustress, is pivotal for governing biological processes and physiological functions including cell cycle and proliferation, circadian clock, innate immunity, self-renewal of stem cells and neurogenesis.^103–106^ However, a variety of stimuli, including nutrient perturbation, genotoxic stress, infection, pollutants and xenobiotics, trigger ROS overproduction, resulting in excessive oxidant challenge (oxidative distress). Oxidative distress causes damage to fundamental macromolecules including proteins, lipids, RNA and DNA at a cellular level, which promotes abnormal cell death and inflammation, often culminating in additional oxidative stress at a systemic level.^95,107^ Through giving rise to fast, barrier-less and non-selective oxidation reactions that are responsible for a severe insult of both cell and systematic tissues, oxidative stress is involved in a myriad of pathologies. Of note, NAD^+^ deficiency exerts effects on the emergence of oxidative stress in multiple diseases, while boosting NAD^+^ has protective effects due to enhancement of antioxidant capacity via increasing the GSH levels and the activity of antioxidant enzymes.^108^ To counteract the detrimental effects of oxidants, cells can heighten the production of reducing equivalents such as NADPH.^109^ Moreover, NAD^+^-consuming enzymes, such as SIRT3, can also manipulate the cellular redox status via regulating the activity of enzymes for ROS generation and antioxidant factors for ROS eradication.^110–112^ Therefore, NAD(P)^+^/NAD(P)H represents the switching hub that controls prooxidant-antioxidant balance and determines the redox biology (Fig. 3).

#### NADH/NADPH as the electron donor in ROS generation

Major endogenous ROS via superoxide radicals is constantly produced by both non-enzymatic reactions such as the mitochondrial respiration that needs NADH and enzyme-catalyzed reactions including NOXs that require NADPH.^113^

In a physiological context, the vast majority of cellular ROS is produced in mitochondria using NADH as electron donor.^114,115^ Mitochondrial NADH supplies NADH dehydrogenase (complex I) with electrons, which along with the electrons obtained from FADH_2_ via complex II drive the mitochondrial ETC to generate a proton (H^+^) gradient across the IMM for the production of ATP. The complex I and complex III of ETC are able to produce the superoxide anion radical (O_2_^–^) and release it to both the matrix and the intermembrane space.^114–116^ Additionally, NADH or FADH_2_ is the electron carrier for the mitochondrial membrane proteins, such as GPDM and FQR, and the metabolic enzymes in matrix, such as OGDH and PDH, all of which contribute to ROS production.^115^

Another significant intracellular source of ROS is the NOXs, especially in response to physiological stimuli, including growth factors, hormones and cytokines, and pathological impulse, such as bacterial and viral infections.^114^ Rather than generating ROS as a by-product, NOXs produce superoxide as a primary product using NADPH as the electron donor.^117^ The NOX proteins, including NOX1-5 and DUOX1/2, have the conserved NADPH-binding site at the C-terminus, which extracts electrons from NADPH. The presence of FAD-binding region and transmembrane hemes enable NOXs to act as an electron-transportation chain that transfers two electrons from cytosolic NADPH to extracellular O_2_, resulting in the generation of O_2_^–^.^95,116^

Beyond mitochondria and the NOX family, a variety of enzymes including xanthine oxidase (XO), NOS, lipoxygenase and cytochromes P450 (CYP) can all produce ROS using NAD(P)H as electron donor.^115^ Mammalian xanthine oxidoreductase (XOR), one enzyme in purine catabolism, can exist in both dehydrogenase (XDH) form, which prefers NAD^+^ as the electron acceptor, and XO form, which transfers the electrons directly to O_2_, leading to the formation of O_2_^–^ and H_2_O_2_.^118,119^ Receiving electrons from NADPH, NOS catalyzes the production of NO from L-arginine participating in a number of biological processes, including neurotransmission, vasodilation and immune response.^120,121^ ROS are also produced via the metabolism of arachidonic acid by lipoxygenases in the presence of NADH or NADPH.^122–124^ Mammalian CYPs are a family of heme-containing NAD(P)H-dependent monooxygenases that metabolize numerous endogenous metabolites, including fatty acids and steroids, and exogenous substrates, including carcinogens, pesticides and drugs, resulting in continuous production of ROS.^115,125,126^

#### NADPH as the final reducing power for antioxidant defense

Besides functioning as the electron donor for ROS production, NADPH also supplies the reducing power for antioxidant defense. To fine-tune the redox homeostasis that can either prohibit the damage by oxidative distress or maintain the physiologic ROS to sustain normal cellular processes, organisms have evolved a complex antioxidant defense system consisting of both enzymatic and non-enzymatic scavengers.^106,127,128^ Intriguingly, both enzymatic and non-enzymatic antioxidant system components exhibit their effects in coordination with each other to contribute to redox homeostasis and cell fate using NADPH as the ultimate donor of reductive power.^116,129,130^ Two classes of enzymatic components, glutathione reductases (GRs) and thioredoxin reductases (TrxRs), are homologous flavoenzymes that use electrons from NADPH to reduce a disulfide to a dithiol. Then, the active site dithiol in GRs reduces the oxidized GSH (the disulfide GSSG) into reduced GSH, the most important non-enzymatic scavenger. GSH is able to reduce the disulfide bonds and hydroperoxides by glutathione peroxidases (GPxs) or promote the glutathionylation at cysteine residues by Glutathione S‐Transferase (GST) to protect protein from oxidation.^107,131–133^ Similarly, mammalian TrxRs maintain the reduced thioredoxin (Trx) concentration that supports peroxiredoxin (Prx) to remove H_2_O_2_.^94,134^ Therefore, through supplying electrons for bioreductive synthesis and the regeneration of GSH and reduced thioredoxin, NADPH plays critical roles in the maintenance of redox homeostasis and modulating redox signaling.^133^

#### NAD^+^-dependent enzymes control redox homeostasis

SIRT3 acts as an essential modulator of oxidative stress via deacetylation of substrates associated with both ROS generation and detoxification. SIRT3 deacetylates and activates the multiple components of ETC including NDUFA9 of Complex I, SDHA of Complex II and core I subunit of complex III. The alteration of ETC, therefore, might contribute to an increased ROS generation.^135–137^ SIRT3 also enhances cellular antioxidant capacity through augmenting the reducing power, NADPH, and increasing the activity of antioxidant enzymes, such as SOD2 and catalase. SIRT3-mediated deacetylation of IDH2 increases the generation of mitochondrial NADPH, which elevates the reduced GSH levels.^138^ Simultaneously, SIRT3 can not only induce the expression of antioxidant enzymes by activating the FOXO3a, but also enhance the activation of SOD2 and catalase via NAD^+^-dependent deacetylation.^139,140^ Besides SIRT3-mediated deacetylation, SIRT5-dependent desuccinylation improves ROS detoxification via increasing SOD1 activity.^141^ These findings reveal a new redox regulation of NAD^+^ by SIRT3‐dependent deacetylation in response to oxidative stress, improving the resistance to the detrimental effects of oxidative damage.

### NAD^+^ sustains genomic stability

**Fig. 4:**
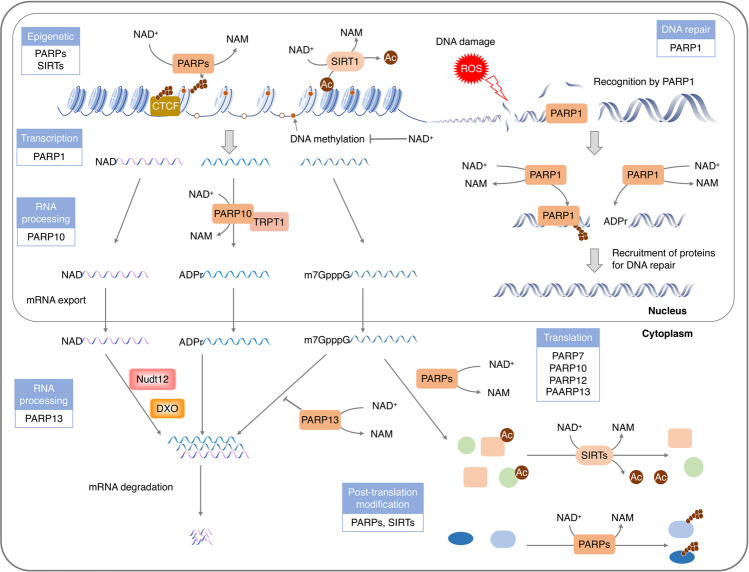
NAD^+^ serves as a pivotal regulator of gene expression. NAD^+^ and its metabolites are used as substrates and cofactors for reactions that coordinate genomic stability, epigenetic status and RNA processing through NAD^+^-dependent enzymes. NAD^+^-dependent histone-deacetylases, especially SIRT1, possess deacetylase activities on multiple transcription coactivators as well as histones, resulting in epigenome remodeling. The lower activity of sirtuins upon lower level of NAD^+^ may contribute to histone hyperacetylation and aberrant gene transcription. Using NAD^+^ as a (ADP)-ribose donor, PARPs mediate ADP-ribosylation on itself or on a variety of nuclear target proteins such as topoisomerases, DNA polymerases, histones and DNA ligases, playing roles in genome stability and gene regulation, from chromatin to RNA biology. Recently, it has been found that NAD^+^ can also serve as a nucleotide analog in DNA ligation and RNA capping in response to stresses. Abbreviations: CTCF, CCCTC-binding factor

The constant challenges from endogenous ROS/RNS or exogenous insults, such as radiation, chemical mutagens and carcinogens, render the DNA damage a relatively common cellular event. Notably, DNA damage and subsequent genome instability are major driving forces for tumorigenesis and aging via driving mutation. To sustain the genome stability, cells have evolved a complicated fine-tuning mechanism, termed as DNA-damage response (DDR), to detect and repair DNA lesions.^142–145^ As key regulators of multiple DNA repair pathways, PARPs and sirtuins modulate the post-modification of repair components using NAD^+^ as co-substrate (Fig. 4). Consistently, NAD^+^ deficiency leads to an impaired DDR and an increased genomic instability, suggesting an interplay between genomic stability and NAD^+^ metabolism.^145–147^

#### DNA ligation

The pathological DSBs are primarily repaired by the NHEJ, a process involves synapsis, end-processing and ligation.^148,149^ DNA ligases-mediated DNA end ligation is initialized by adenylating the ligase with an AMP moiety. In prokaryotes, both ATP and NAD^+^ are adenylation donor for DNA ligases, while in eukaryotes, only ATP is known to be used by DNA ligases for the adenylation.^150^ Recently, it has been reported that human DNA ligase IV, a crucial enzyme in NHEJ, can acquire AMP moiety from NAD^+^ for DNA end ligation. The BRCA1 C-terminal (BRCT) domain of ligase IV is required to recognize of NAD^+^ for subsequently ligation in NHEJ.^7^ Although future studies will be required to fully characterize the structure of NAD^+^-associated human DNA Ligase IV, these findings reveal that like ATP, NAD^+^ can serve as a provider of adenylation for DNA ligation in the NHEJ DNA repair pathway.

#### DNA repair

Beyond regulating the NHEJ pathway acting as an adenylation donor, NAD^+^ also modulates other DNA repair pathways via activating NAD^+^-consuming enzymes, PARPs and sirtuins.^151,152^ As sensitive DNA damage sensors, PAPRs are recruited and immediately activated by DNA breaks. The DNA-bound PARPs, such as PARP1-3, can attach the mono-ADP-ribose (MAR) or poly-ADP-ribose (PAR) moieties directly to the DNA breaks.^153–156^ Meanwhile, PARPs also catalyze the ADP-ribosylation of various proteins that facilitate the chromatin relaxation and the recruitment of repair factors.^157–162^ The effect of PARPs to stimulate chromatin decompaction might be exerted via the steric hindrance of PAR chain, the negative charge of DNA and PAR, or the displacement of core histones.^162,163^ Simultaneously, the accumulated PARs at DNA break sites are required for the recruitment of DNA repairs, including XRCC1, DDB2, ALC1, RECQ1, CHD2, BRCA1, Ligase V, MRE11 and NBS1, to initiate DNA repair.^164–167^ Similarly, DNA damage induces the relocalization of NAD^+^-dependent deacetylase SIRT1 to DNA breaks, which promotes DNA repair via opening the chromatin and recruiting the main DNA repair factors including KU70, NBS1, WRN, KAP1, XPA and APEX1.^168–177^ Additionally, PARPs and sirtuins also simulate genomic damage-signaling kinases, including ATM, p53, DNA-PK, CIRBP and FOXOs, to accelerate DNA repair.^178–182^

Given that DNA damage-activated PARPs account for up to 90% of cellular NAD^+^ consumption, the DNA repair activity is highly dependent on the cellular NAD^+^ concentration.^133,183,184^ As expected, decreased NAD^+^ levels induce the accumulation of DNA damage, whereas replenishing intracellular NAD^+^ stimulates the DNA repair.^185–187^ In contrast to the positive effect of NAD^+^ on DNA repair, NADP^+^ suppresses the ADP-ribosylation-mediated DNA damage repair via functioning as an endogenous inhibitor of PARPs. The structure of NADP^+^ similar to that of NAD^+^ renders its binding to PARPs. However, NADP^+^ has an additional phosphate group on the 2’ position of the ribose ring, which abolishes the formation of linear PAR chain.^184^

### NAD^+^ manipulates the gene expression

Cellular metabolism, such as NAD^+^ metabolism, is directly connected to gene expression through regulating the post-translational modifications (PTMs) of histones, the covalent chemical modifications of DNA, the activity of transcription factor and RNA processing.^188,189^

#### Histone modification

Histone modification is one of the most crucial epigenetic modification that affects DNA structure and gene expression. The post-translational modifications of histones include acetylation, ADP-ribosylation, phosphorylation and methylation. Among these modifications, the acetylation and ADP-ribosylation are regulated by NAD^+^-dependent enzymes, sirtuins and PARPs, respectively. Sirtuins, also known as NAD^+^-dependent class III HDACs, remove the acetyl groups from histone, which restores the electrostatic affinity between DNA and histones to stabilize the chromatin architecture.^190,191^ SIRT1-3 maintain the chromatin structure via deacetylation of a crucial histone residue, H4K16. The reduced intracellular NAD^+^ concentration limits the deacetylase activity of SIRT1, resulting in elevated H4K16Ac and gene expression.^192,193^ SIRT6 can coordinate NF-κB to deacetylate the H3K9Ac, sequestering the expression of glucocorticoid receptors (GRs).^194^ SIRT7 is able to selectively deacetylate the H3K18Ac, which represses the expression of a specific set of gene targets that is linked to oncogenic transformation.^195^ Histones also serve as acceptors of ADP-ribose upon DNA damage to initiate DNA repair.^183^ The ADP-ribosylation of histones by PARP-1 induces the dissociation of nucleosomes, leading to the decompaction of chromatin. Furthermore, PARP-1-mediated PARylation of KDM5B prevents the demethylation of H3K4me3, rendering the exclusion of H1 and the opening of chromatin. The decompensation of chromatin structure, therefore, allows the loading of the transcriptional machinery and facilitates gene transcription.

#### DNA modification

DNA methylation is another widely studied epigenetic modification that is often involved in the regulation of gene expression. NAD^+^ deficiency can promote the DNA methylation, resulting in gene silencing. NAD^+^ depletion elevates the methylation of BDNF promoter, triggering the dissociation of the DNA methylation-sensitive nuclear factor CCCTC-binding factor (CTCF) and cohesin from the BDNF locus. The detachment of these factors causes the altered methylation and acetylation of histone at this locus, leading to chromatin compaction and gene silencing.^196^ The NAD^+^-consuming enzymes, PARPs, are associated with the regulation of DNA modification. Inhibition of the PARPs-mediated ADP-ribosylation causes a chromatin compaction DNA hypermethylation.^197^ PARP-1 can be activated by the chromatin insulator CTCF even without niched DNA and efficiently automodified, dependent on NAD^+^. The PARs of PARP‐1 compete with DNA for the noncovalently binding DNMT1, causing suppression of its methyltransferase activity.^198,199^ Therefore, the NAD^+^-dependent enzymatic activity of PARP-1 is a crucial regulator of gene expression via protecting the genome from aberrant hypermethylation.

Another evidence linking the NAD^+^ metabolism with DNA methylation is the NNMT, which transfers the methyl group from SAM to NAM, producing S-adenosylhomocysteine (SAH) and a stable metabolites 1-methylnicotinamide (MNA). Given that the SAM is the universal methyl donor for methylation of substrates including proteins, nucleotide acids and lipids, the NNMT induced methyl sink in the form of MNA impairs the genome methylation.^200,201^ Moreover, the methionine metabolism catalyzed by NNMT diverts the NAM from the NAD^+^ salvage pathway. As a consequence, the reduced cellular NAD^+^ content restricts the PARP1-catalyzed ADP-ribosylation and the following DNA methylation. Collectively, increased NNMT expression inhibits the DNA methylation through not only decreasing the cellular NAD^+^, the donor for ADP-ribosylation required for methylation, but also reducing the level of SAM, the methyl donor for methylation.

### NAD^+^ modulates RNA processing

#### Decorating of RNA as a nucleotide analog

Chemical modifications of the RNA 5’-end play a pivotal role in various biological functions including the protection of RNA from exonuclease cleavage, the recruitment of proteins for pre-mRNA processing and nuclear export and the initiation of protein synthesis. Recently, NAD^+^ has been found to be incorporated into RNAs as an initiating nucleotide during transcription to form NAD^+^ cap in different organisms including bacterial, yeast, human and virus.^8–12,202^ Unlike the well-characterized m^7^G-capped mRNA, which maintains highly stability of mRNA for translation, the NAD^+^-capped RNAs are vulnerable to decay and are inefficiently translated in cells.^9,10,203^

NAD^+^ capping can be catalyzed by eukaryotic nuclear RNAP II using NAD^+^ and NADH as non-canonical initiating nucleotides (NCINs) in de novo transcription initiation.^8^ Besides the RNAs produced by nuclear RNAP II, RNAs synthesized by mitochondrial RNAP (mtRNAP) can also be NAD^+^ capped. The human mtRNAP conducts a higher efficiency of NAD^+^ capping than the nuclear RNAP II, leading to ~15% NAD^+^ capping of mitochondrial transcripts.^204^ The 5′ end NAD^+^ cap of RNAs in the cytoplasm will be removed by two mammalian hydrolases, DXO and Nudt12. Reported as a deNADding enzymes, the Nudt12 contributes to the decapping of RNA following exposure to nutrient stress, such as glucose deprivation, while DXO is responsible for the environmental stress, such as heat shock.^12,205–207^ In line with that NAD^+^-capped RNA levels respond to the cellular total NAD^+^ concentrations, the capping efficiencies of NAD^+^ capping and NADH capping are also regulated by intracellular NAD^+^/NADH ratio.^204,207^ These results raise the possibility that RNAP II and mtRNAPs might function as both sensors and executors, which sense NAD^+^/NADH ratios and induce the NAD^+^ capping to regulate the gene expression, leading to the crosstalk between cellular NAD^+^ metabolism and transcriptional activity.

#### ADP-ribosylation of RNA

Beyond decorating RNA as a nucleotide analog, NAD^+^ also provides the ADPR groups for the reversible mono-ADP-ribosylation of RNA phosphorylated ends. This RNA modification is catalyzed by PARP10 with a preference for 5′ ends, depending on NAD^+^ concentration. In addition to PARP10, TRPT1, a PARP homolog, also catalyzes the ADP-ribosylation of RNA. The ADP-ribosylation renders RNA resistant to phosphatase, which might protect the RNA from the nuclease attack. Similar to the reversible ADP-ribosylation of proteins and DNA, the ADP-ribosylation of RNA can also be efficiently reversed by several cellular hydrolases including TARG1, MACROD1-2, PARG, NUDT16 and ARH3 viruses.^29^ Besides human hydrolases, macrodomain-containing hydrolases from VEEV and SARS can remove the ADP-ribosylation of RNA catalyzed by PARPs, suggesting a potential mechanism of pathogenesis via inhibiting the antiviral activity of IFN-stimulated genes, PARPs. Altogether, the ADP-ribose moieties attached to RNA end might protect RNA against degradation or serve as a platform for recruiting proteins, controlling the functional state of RNA.

### NAD^+^ facilitates cellular energy metabolism

#### NAD^+^/NADH as hydride-donating coenzyme for metabolism

Acting as a coenzyme, NAD^+^ plays pivotal roles in energy metabolism pathways including glycolysis, the TCA cycle, OXPHOS, FAO and alcohol (ethanol) metabolism.^66^ The glycolysis process begins with one glucose molecule and ends with two molecules of pyruvate, which are subsequently transported into the mitochondria to begin the TCA cycle. NAD^+^ promotes glycolysis by facilitating the enzymatic reactions catalyzed by GAPDH and lactate dehydrogenase (LDH), which use NAD^+^ as a coenzyme.^208,209^ NAD^+^ is reduced to NADH coupled with the oxidation of G3P to 1,3-BP by GAPDH.^210^ Cytosolic pyruvate can also be converted to lactate by LDH, coupled with the oxidation of NADH to NAD^+^.^211^ This process helps maintain the cytosolic level of NAD^+^, thus contributing to the continuity of glycolysis. When transported into the mitochondria, the glycolytic end-product pyruvate is decarboxylated to produce acetyl-CoA by PDH complex, which reduces NAD^+^ to NADH simultaneously.^210^ Acetyl-CoA then starts the TCA cycle, where NAD^+^ serves as a coenzyme for three rate-limiting enzymes, α-ketoglutarate dehydrogenase (KGDH), isocitrate dehydrogenase 3 (IDH3) and malate dehydrogenase (MDH2), to generate NADH. Thus, the TCA cycle can convert four molecules of NAD^+^ to NADH using one molecule of pyruvate in the mitochondria under aerobic conditions.^212^ As an electron donor, NADH produced in the TCA cycle plays a crucial role in ATP synthesis by OXPHOS, which generates most of the energy through the H^+^ gradient in animal cells.^213^

FAO breaks down a long-chain acyl-CoA, which is generated from fatty acid and coenzyme A by acyl-CoA synthetase in the cytosol, to generate acetyl-coA, NADH and FADH_2_ in the mitochondria.^214^ This process is performed in repeated cycles, each of which removes a two-carbon acetyl-coA from the acyl-CoA via four enzymes, the enoyl-CoA hydratase, ketoacyl-CoA thiolase, acyl-CoA dehydrogenase (ACADs) and hydroxyacyl-CoA dehydrogenase (HADH). The last cycle generates two molecules of acetyl-coA. FADH_2_ is generated by ACADs, while NADH is produced from NAD^+^ in the reaction catalyzed by HADH. Both NADH and FADH_2_ generated in the FAO are utilized to synthesize ATP by the ETC. NAD^+^ is also a cofactor in alcohol oxidation metabolism taking place mainly in liver cells. Alcohol oxidation is completed in a two-step reaction by two enzymes, alcohol dehydrogenase (ADH) and aldehyde dehydrogenase (ALDH), which catalyzes the reduction of NAD^+^ to NADH.^215^ Both the sufficient glycolysis and the effective oxidation of alcohol require fast reoxidation of NADH to NAD^+^ through the coordinated reduction of pyruvate to lactate by LDH or production of ATP by mitochondrial ETC.^213,216^

#### NAD^+^-dependent modification of metabolic enzymes

Beyond serving as a hydride-donating coenzyme for metabolism, NADH/NAD^+^ also acts as co-substrate for the sirtuins-mediated post-translational modification of metabolic enzymes including acetylation, ADP-ribosylation, succinylation and malonylation. A large number of enzymes that participated in cytosolic glycolysis, gluconeogenesis, the urea cycle, nitrogen metabolism, glycogen metabolism, mitochondrial fatty acid oxidation, the TCA cycle and amino acid catabolism can be regulated by sirtuins.^217,218^

The mitochondrial sirtuin-related acetylome covers almost all the mitochondrial metabolism, including the enriched SIRT3-related TCA cycle, ETC, FAO, the SIRT4-associated anion transporters, the translation and energy metabolism, the SIRT5-regulated TCA cycle and branched chain amino acid catabolism (BCAA) metabolism.^219,220^ The mitochondria SIRT3 function as a metabolic sensor that links the cellular energy status with the mitochondrial protein acetylation patterns. In healthy mitochondria, SIRT3 interacts with ATP5O, while the low pH owing to the loss of membrane potential weakens the binding affinity between SIRT3 and ATP5O, leading to the redistribution of SIRT3 to other mitochondria substrates. The pH-insensitive association between SIRT3 and ATP5O provides a fundamental role for SIRT3 in resetting mitochondrial acetylation in response to stress.^219^ The transition to fasting enhances both the cellular NAD^+^ level and the SIRT3 expression, which, in turn, catalyzes the deacetylation of LCAD to promote fatty-acid oxidation.^221^ SIRT3 orchestrates the metabolism reprogramming via controlling the balance between cytosolic glycolytic metabolism and mitochondrial oxidative metabolism.^222^ SIRT3 also plays a regulatory role in proline metabolism via deacetylation of PYCR1.^223^

SIRT4 modulates mitochondria energy homeostasis and longevity based on its lysine deacetylase, lysine deacylase, lipoamidase and ribosylase activity. Under nutrient-replete conditions, the deacetylation of malonyl CoA decarboxylase (MCD) by SIRT4 plays a pivotal role in lipid homeostasis via suppressing fatty acid oxidation and inducing lipid anabolism.^224^ The lysine deacylase activity of SIRT4 is involved in the control of leucine metabolism and insulin secretion through regulating the acylation status of enzymes in these pathways.^225^ SIRT4 also acts as a cellular lipoamidase with a preferred catalytic efficiency for lipoyl- and biotinyl-lysine modifications to its deacetylation activity. SIRT4 hydrolyzes the lipoamide cofactors from the E2 component dihydrolipoyllysine acetyltransferase (DLAT), leading to diminished PDH activity.^226^ Furthermore, SIRT4 uses NAD^+^ to ADP-ribosylate and reduce GDH activity, thereby inhibits insulin secretion in response to amino acids in β cells.^227^

SIRT5 is an NAD^+^-dependent lysine desuccinylase, demalonylase, and deglutarylase.^228^ BAT specific deletion of Sirt5 exhibits hypersuccinylation of proteins involved in the amino acid metabolism, ETC and FAO. A bunch of mitochondrial proteins have succinylation modification, such as UCP1 in thermogenic function, GLUD1 in glutamate metabolism, SDH in ETC, the TCA cycle, GLS2 and CPS1 in glutaminolysis, ECHA and VLCAD in FAO, HMGCS2 in ketogenesis and SHMT2 in serine catabolism.^229–233^ SIRT5-mediated desuccinylation also participates in protection against peroxisome-induced oxidative stress via targeting ACOX1.^230^ Moreover, SIRT5 functions as a leading regulator of protein malonylation in both cytosolic metabolisms including glycolysis, gluconeogenesis and mitochondria FAO. For instance, SIRT5 increases the activity of GAPDH by demalonylation, thereby controlling the energetic flux through glycolysis.^227,234,235^ Collectively, sirtuins orchestrate an integrated modulation of metabolic pathways via NAD^+^-dependent post-translational regulation in response to diverse nutrient signals.

### Rhythmic NAD^+^ oscillates circadian clock

Organisms have developed internal clocks as a timekeeping mechanism to collaborate biological processes with the exogenous environmental and endogenous factors. NAD^+^ functions as a metabolic driver of circadian transcription via epigenetic mechanisms, transducing signals originated by environmental stimuli to the circadian clock. The linkage of NAD^+^ metabolism to the internal clocks is firstly evidenced by that the NAD(P)^+^/NAD(P)H ratio modulates the DNA-binding activity of the core oscillators, such as CLOCK: BMAL1 and NPAS2: BMAL1 heterodimers. The redox state of FAD and NADPH also displays an oscillation pattern in organotypic slices of suprachiasmatic nucleus (SCN).^236^ The circadian control of intracellular NAD^+^ levels by the clock is attributed to the oscillation expression of NAMPT, a rate-limiting enzyme in the salvage of NAD^+^ with a 24-hour rhythm.^36,38,237–239^ The E-boxes in the promoter of *Nampt* gene allow the direct transcriptional control by the CLOCK: BMAL1 chromatin complex.^240^ Furthermore, the expression of enzymes in the NAD^+^ salvage pathway, including Nmrk1, Nampt, and Nadk, has circadian oscillation patterns in WT and Liver-RE mice that exclusively express BMAL1 in the liver, suggesting the circadian clock might reprogram NAD^+^ salvage synthesis to maintain the fluctuation of NAD^+^.^241^

The oscillation of NAD^+^, in turn, coordinates the transcription and behavior through the circadian clock. The reduction of NAD^+^ in old mice dampens the circadian transcription, which can be rescued by NAD^+^ repletion to youthful levels with NR.^242^ The regulatory effect of NAD^+^ on circadian reprogramming is mediated by changing the activity of sirtuins and PARPs, which determines the transcriptional activity of core oscillators. SIRT1/6 can be recruited into the core clock CLOCK: BMAL1 complex, which renders the rhythmic acetylation of BMAL1 and the cyclic H3K9/14Ac at circadian promoters on their target genes.^38,238,243^ Besides, the oscillation activation of SIRT1 also regulates the circadian dynamics via deacetylation of the core clock repressor PER2^K680^ and mixed-lineage leukemia 1 (MLL1), thereby controlling rhythmic chromatin property and the activity of BMAL1: CLOCK complex.^36,38,238,242,244^ Similar to sirtuins, the activity of PARPs is also regulated by the circadian clock. The oscillation activation of PARP-1 interacts with and poly(ADP-ribosyl)ates CLOCK, leading to suppressed binding of CLOCK: BMAL1 to DNA and altered circadian gene expression.^245^ Moreover, PARP1 interacts with CTCF in a circadian manner, regulating lamina-associated chromatin and circadian oscillations in transcription.^246,247^ These reports indicate a connection between NAD^+^-dependent epigenetic modification and the core circadian clockwork circuitry.

The interplay of NAD^+^/NADP^+^ metabolism with circadian clock is further evidenced by the oscillating redox, in which ROS levels display a different liver pattern compared to other tissues due to the unique NAD^+^ oscillation in response to the autonomous hepatic clock. Circadian disruption in beta-Bmal1(-/-) mice and arrhythmic Clock^Δ19^ mice decrease the Nrf2 expression and subsequently impair the antioxidant defense system, contributing to increased ROS accumulation, oxidative damage and mitochondrial uncoupling.^248,249^ Prxs, the most critical H_2_O_2_-removing enzymes, exhibit rhythmic cycles of oxidation.^250^ The circadian clock system can also regulate the production and consumption of GSH through circadian regulation of the rate-limiting enzymes in GSH biosynthesis and cellular detoxification.^236^ The oxidation cycle of both Prxs and GSH is directly influenced by the availability of redox cofactor NADPH, suggesting that NADPH metabolism might play a vital role in controlling redox rhythmic and transcriptional oscillations. In line with this notion, it has been demonstrated that inhibition of NADPH production from PPP alters circadian rhythms through changing the activity of CLOCK: BMAL1.^251–253^ Thus, NAD(P)^+^/NAD(P)H acts as an important modulator of cellular energetic status, enabling the reset of redox rhythmic and transcriptional oscillations based on metabolic signals.^254^

### NAD^+^ metabolism programs immunity and inflammation

NAD^+^, along with citrate and succinate, is a novel class of metabolites with inflammatory signaling capacity, linking the NAD^+^ metabolism to the programming of immune responses.^255^ Restoring the NAD^+^ levels via de novo biosynthesis in the liver prevents hepatic lipid accumulation and attenuates inflammation in mice on a high-fat diet (HFD).^15^ Similarly, increased generation of NAD^+^ via the KP in resting, aged or immune-challenged macrophages restores OXPHOS and homeostatic immune responses, whereas inhibition of de novo NAD^+^ synthesis induces an increased inflammation-associated TCA-cycle metabolite succinate and elevated mitochondria-generated ROS, resulting in rising innate immune dysfunction in aging and age-associated diseases.^256^ Mitochondrial complex III produces ROS immediately after stimulation, which has an essential role in inflammatory macrophage activation. However, the mitochondrial ROS are also responsible for DNA damage, which causes the abundant consumption of NAD^+^ by PARPs. The NAD^+^ abundance as well as the NAD^+^/NADH ratio, therefore, decline significantly even with the induction of the de novo synthesis from the KP in response to the lipopolysaccharide (LPS) challenge.^256,257^ To maintain the cellular NAD^+^ level, NAD^+^ salvage enzyme NAMPT has been activated by LPS to boost the salvage pathway.^258^ Elevated expression of NAMPT maintains the NAD^+^ content to drive the glycolysis, which supports the activation of inflammatory macrophages.^258^ While in the mitochondrial respiration-impaired cells, NAD^+^ could reduce the exacerbated inflammatory response via improving lysosomal function. The addition of nicotinamide precursor NAM in mitochondrial respiration-impaired cells restores the lysosomal function and limits the increased proinflammatory profile.^259^ Furthermore, endotoxin dose-dependent switch of NAD^+^ biosynthesis pathways from NAMPT-dependent salvage to IDO1-dependent de novo biosynthesis maintains the nuclear NAD^+^ pool, which promotes SIRT1-directed epigenetic regulation of immune tolerance.^260,261^

Owing to its rate-limiting enzymatic activity in NAD^+^ salvage pathway, the elevated expression of NAMPT in the innate immune cells, including macrophages and dendritic cells, further proposed a link between intracellular NAD^+^ levels and inflammation.^262–264^ Specific competitive inhibitor of NAMPT could ameliorate the immunity or inflammatory disorders, including DSS-induced colitis, arthritis via reducing intracellular NAD^+^ levels in inflammatory cells and circulating inflammatory cytokines, including IL-1beta, TNF-alpha, and IL-6.^265–267^ Cellular levels of NAD^+^ regulated by NAMPT also impacts NAD^+^-dependent enzymes, such as sirtuins. For example, sirtuins modulated the optimal TNF translation.^268^ The elevated NAD^+^ levels concomitant with SIRT1 switches the NF-κB-dependent transcription into the RelB-dependent transcription of the TNF-α in endotoxin tolerant sepsis blood leukocytes.^269^ Additionally, SIRT6 can modulate TNF production by regulating the TNF mRNA translational efficiency.^269^ In a pancreatic cell line, SIRT6 induces the production of cytokines including IL‐8 and TNF, which promote cell migration.^26^ Sirtuins control immune responses via directly regulating inflammatory transcription factors, including deacetylation of FOXP3 to repress Treg cell responses, deacetylation of RORγt to promoteT_H_17 cell responses, and suppression of NF-κB to reduce inflammatory responses.^188^

Besides NAD^+^, NADPH also plays essential roles in immunity and inflammation, mainly dependent on the NADPH oxidases and redox signaling.^270^ In an inflammatory response, activation of epithelial and immune cells triggers NOXs to generate ROS, which can directly kill microorganisms.^271–273^ NOXs-derived ROS can also act as a second messenger in signaling transduction. It has been reported that NOX4 directly interacts with TLR4, which is pivotal for LPS-mediated NF-κB activation.^274^ In the nasal airway epithelium, the interaction of TLR5 and another NOX isozyme, Duox2, induces the ROS generation and IL-8 expression in response to flagellin exposure.^275,276^ The phagocytic NADPH oxidase complex can also be activated by Rubicon to induce a ROS burst, inflammatory cytokine production and potent antimicrobial activities.^277^

## Abnormal NAD^+^ metabolism in the pathophysiological condition

Given the essential regulatory role of NAD^+^ in fundamental physiologies, NAD^+^ metabolic abnormalities contribute to the pathophysiology of various diseases, such as infection, cancers, metabolic diseases, aging and age-associated neurodegeneration disorders.

### Perturbed NAD^+^ metabolism in response to infection

**Fig. 5:**
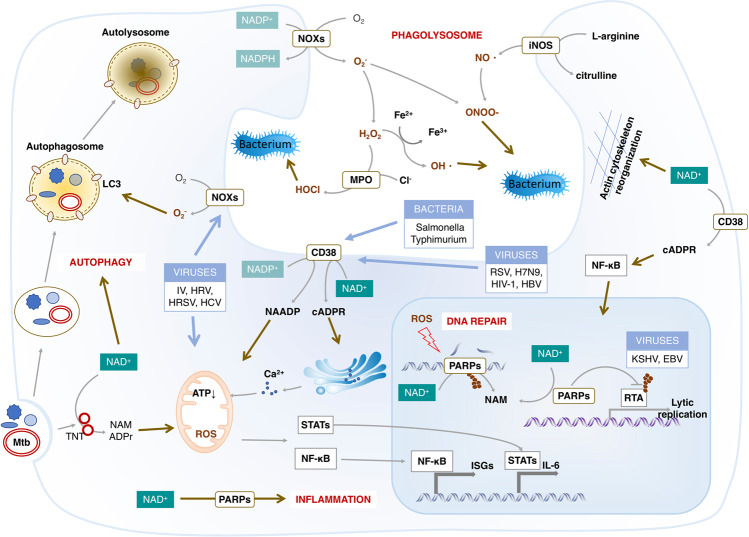
Physiological actions of NAD^+^ in the host response to infection. Microbial infection, including viruses and bacteria, causes oxidative stress that has a critical effect on both the microbe and host cells. The production of ROS from NOXs depending on NADPH termed respiratory burst is a powerful antimicrobial weapon and a major component of the innate immune defense against bacterial and fungal infections. Meanwhile, oxidative stress causes the host DNA damage that enhances the consumption of NAD^+^ by elevated PARPs. The intracellular NAD^+^ can also be reduced by activation of CD38 that is required for the inflammation against infection. The NAD^+^ deficiency therefore might not be able to support the clearance of microbial by autophagy or phagolysosome, the innate immune and inflammation response. Abbreviations: EBV, Epstein-Barr virus; HCV, hepatitis C virus; HRV, human rhinovirus; HRSV, human orthopneumovirus; iNOS, inducible nitric oxide synthase; ISGs, interferon-stimulated genes; IV, Influenza virus; KSHV, Kaposi’s sarcoma-associated herpesvirus; MPO, myeloperoxidase; Mtb, Mycobacterium tuberculosis; NOXs, NADPH oxidases

Microbial infection, including viruses and bacteria, causes an imbalance in the cellular redox environment, thus inducing different responses, e.g., antioxidant defenses, cell signaling, immune response and other processes. NAD^+^ or NADPH level determines the role of ROS in infections, either protecting against invading microorganisms or causing tissue damage during the resulting excessive inflammation (Fig. 5).

#### NAD^+^ mitigates viral infection-induced oxidative damage

Oxidative stress is implicated as a pathogenic factor in viral infection.^278^ It can be caused by diverse virus families ranging from DNA (i.e., HBV, EBV, HSV-1) to RNA viruses (i.e., HCV, RSV, DENV, Influenza, ZIKA, HIV).^279–283^ The increased cellular ROS by viral infection cause DNA damage, gene mutation, cell death, viral DNA integration and tumorigenesis.^284–290^ For instance, acute phase of HCV infection induces oxidative stress via enhancing NOXs expression and activity to generate ROS generation and decreasing GSH, which supports the high rates of viral replication and apoptotic cell death. On the other hand, the chronic infection maintains a reduced environment to establish viral persistence.^291^ Moreover, NOX-induced ROS play various roles in the mechanisms of oncogenesis by HCV, including genome instability, epigenetic regulation, inflammation and modulation of cell growth and death.^292^ In RSV-infected airway epithelial cells, NOX-generated ROS trigger the activation of the transcription factors IRF and STAT, thereby inducing the expression of chemokines and cytokines involved in the immune/inflammatory responses of the lung.^293^ NQO1, an enzyme involved in the elimination of ROS, is inhibited by HBx, leading to decreased GSH levels and increased susceptibility of hepatoma cells to oxidative damage, cumulating in HBV-associated pathogenesis of chronic liver diseases.^294^

To repair the oxidative stress-induced DNA damage, a large amount of NAD^+^ were consumed by elevated PARPs in response to virus infection, i.e., HSV-1, ZIKV and New Sindbis virus (SV).^281,282,295^ Beyond the important role in DNA repair, PARP-1 also acts as a modulator of NF-κB, inducing the downstream CCL2-CCR2 signaling, which is required for the recruitment of NK cell to the infection site and viral control.^296^ Therefore, PARPs have antiviral activity against multiple classes of viruses, including retroviruses, alphaviruses, filoviruses, herpesviruses, adenoviruses and coronavirus through enhancing the innate immunity.^297–300^ Sirtuins are another class of NAD^+^-consuming enzyme, which have broad-range antiviral properties on diverse viruses including HCV, HIV-1, HCMV and influenza A (H1N1) virus.^301–305^ Besides controlling the virus replication, PARPs or sirtuins also contributes to the oncogenic virus infection, such as oncogenic gamma herpesviruses KSHV and Epstein Barr virus (EBV) infection, and the tumorigenesis through the epigenetic remodeling.^306–308^ CD38 is the third NAD^+^-consuming enzyme that is upregulated in response to a number of viral infections.^309–312^ CD38 is under the transcriptional control of RSV‐induced IFNs. The CD38‐generated cADPR, in turn, augments the IFNs-induced ISGs and NF-κB-mediated inflammation, leading to the antiviral hyperinflammation response.^311^ In addition to the excessively increased consumption of NAD^+^, multiple viruses cause a decline in NAD^+^ concentration through reducing the protein levels of crucial enzymes in the NAD^+^ biosynthesis pathway, including QPRT and NAMPT.^304,313^ Regarding the redox role of NADH/NADPH in eliminating ROS, the depletion of NAD^+^/NADP^+^ pool exacerbates the oxidative damage during virus infection.^306,308,314–316^

#### NAD^+^ contributes to the bactericidal activity

Bacterial infection induces rapid production of intracellular ROS either by NOXs or mitochondria that are, in turn, crucially required by macrophages to clear bacteria.^105,317^ Elimination of the ROS results in defective bactericidal activity, allowing bacteria to survive and repeatedly colonize various tissue sites.^318,319^ NOXs in immune cells, such as macrophages and neutrophils, are primarily responsible for ROS production and termed respiratory bursts during phagocytic bacterial killing.^320,321^ Additionally, NOX2-generated ROS are necessary for LC3 recruitment to phagosomes, revealing an autophagy-dependent antibacterial activity of NOX2 in phagocytes.^322^ Mycobacterium tuberculosis (Mtb) can trigger the production of ROS via depletion of NAD^+^. TNT, a major cytotoxicity factor of Mtb, hydrolyzes the cellular NAD^+^ to NAM and ADPR, thereby activating the necroptosis effectors MLKL and RIPK3. Moreover, the NAD^+^ depletion or the NAD^+^ hydrolysis products induced signaling contributes to the TNT-triggered ROS production.^323^

Moreover, the NOX-dependent oxidative burst caused by phagocytosis of bacterial cells activates CD38, producing NAADP in the maturing phagosome. NAADP induces the lysosomal Ca^2+^ efflux and calcineurin-mediated TFEB activation, which enhances the expression of pro-inflammatory cytokines including IL-1β, IL-1α and IL-6.^324^ CD38 also exerts bactericidal activity in an NAD^+^-dependent manner.^325,326^ CD38 controls neutrophil chemotaxis to bacterial chemoattractants via producing cyclic ADP-ribose.^326^ In macrophage, high levels of CD38 induced by LXR agonists reduce NAD^+^ levels and interfere with cytoskeletal rearrangements triggered by invasive bacteria, protecting host macrophages from substantial infection.^327^ However, in T-cell, the NAD^+^ depletion by elevated CD38 expression increases the acetylation of EZH2 by in a SIRT1-dependent manner, leading to reduced cytotoxicity of CD8 T cell and enhanced inclination to infections in patients with systemic lupus erythematosus (SLE).^328^ Additionally, the NAD^+^ concentration and NAD^+^/NADH ratio are significantly elevated in response to Group A streptococcus (GAS)-infection. The addition of NAM remarkably enhances the intracellular NAD^+^ content that promotes the autophagosomal acidification and clearance of GAS in endothelial cells.^323^ Therefore, NAD(P)^+^/NAD(P)H exerts the bactericidal activity by promoting the ROS generation, the pro-inflammatory response and the anti-infection autophagy.

### NAD^+^ deficiency accelerates aging

Multiply evidence elucidates that NAD^+^ and NAD^+^-related metabolites govern biological functions in aging, including metabolism, redox homeostasis, mitochondria function and the circadian clock. The NAD^+^ decline during normal aging results in oxidative damage, metabolic disorder, circadian rhythm abnormalities, and mitochondrial dysfunction through regulating signaling pathways, such as p53, NF-κB, PGC-1α and HIF-1α, by sirtuins and PARPs.^329–332^ Accordingly, boosting NAD^+^ provides a therapeutic option for improving the health lifespan and treating aging-related diseases.

#### NAD^+^ deficiency accelerates aging

NAD^+^ levels display steadily reduction in old worms, which causes a further shorter lifespan.^333^ Similarly, mice and rats exhibit an NAD^+^ decline during aging in a variety of tissues, such as muscle, adipose tissue, brain, skin, liver and pancreas.^334^ The reduced NAD^+^ is also observed in the aged human brain and liver.^335^ In line with that, the plasma levels of NAD^+^ and its metabolites, NADP^+^ and NAAD also remarkably decline during aging.^336^

The age-dependent decline of NAD^+^ might be due to either enhanced consumption or reduced biosynthesis. The NAD^+^ levels and NAMPT expression are severely inhibited in various tissues including liver, skeletal muscle, WAT and pancreas in age induced T2D models. The decreased NAMPT might be due to the chronic inflammation and impaired circadian clock during aging.^337,338^ However, another study describes no alteration of the NAMPT mRNA or protein levels in aged mice and human tissues.^52^ Thus, these controversial findings of NAMPT-catalyzed NAD^+^ biosynthesis overage might come from the differential cell type and tissue context, which will be elucidated in future studies. Another explanation for NAD^+^ decline with age is the increased NAD^+^ consumption by PARP or CD38. In contrast to the unchanged levels of PARP1, both the protein levels and enzymatic activity of CD38 are enhanced during aging, contributing to the age-related NAD^+^ decline in mammals. CD38 is also responsible for the mitochondrial dysfunction by regulation of SIRT3 activity.^52^ Nevertheless, CD38-deficient old mice preserve the NAD^+^ levels, mitochondrial respiration and metabolic functions.^339^ CD38 expression might be induced by chronic inflammation, one characteristic during aging.^52,340^

The NAD^+^ decline is a primary driver for the progressive of biological dysfunction and age-related pathologies. Thus, the genetic or pharmacological modulation of NAD^+^ provides a therapeutic option for multiple age-related diseases. Indeed, genetic and pharmacological replenishment of NAD^+^ improves the age-related biologic function and increases lifespan at least in worms and mice.^341,342^ The increased expression of NAMPT in aging human SMCs prolonged lifespan via delaying senescence and enhancing resistance to oxidative stress.^343^ Supplementation with NAD^+^ precursors, NR and NMN, elevated NAD^+^ levels that can maintain the mitochondrial and metabolic functions by activating SIRT1 in mice, leading to an extensive lifespan of mice.^337,344,345^ Augmentation of NAD^+^ by β-lap, a potent substrate of NQO1, effectively prevents ARHL and its accompanying harmful effects by preventing oxidative stress and inflammation and improving mitochondrial function in rodents.^346^ Moreover, mounting evidence has shown that NAD^+^-dependent sirtuins can extend the lifespan of yeast, worms, flies and mice and alleviate many diseases of aging-related pathologies. For instance, both brain-specific or whole-body SIRT1-overexpressing transgenic mice exhibit a slowed aging and a prolonged lifespan.^347,348^

#### Aging-related NAD^+^ decline causes mitochondrial dysfunction

Combining the evidence that mitochondrial dysfunction is a hallmark of aging and NAD^+^ plays a crucial role in the maintenance of mitochondrial function.^341,345^, we can hypothesize that aging-related NAD^+^ decline might be the cause of mitochondria dysfunction. NAD^+^ boosters play a preventive role in aging via the early-phase activated UPR^mt^, and the late-phase induced antioxidant defense. Regulation of NAD^+^ availability by PARP inhibitors and NAD^+^ precursor modulates mitochondrial function through sir-2.1 in worm to extend lifespan.^341^ The PARylation is also markedly increased in muscle and the liver of aged mice in parallel with robustly decline of NAD^+^ levels. Since CSB can limit the activity of PARP1 via displacing it from damaged DNA, in CSB-deficient cells and mice, the PAPRPs-mediated PARylation is increased and accounts for the majority of cellular NAD^+^ consumption. This aberrant activation of PARPs represses the SIRT1 activity and mitochondrial dysfunction, which can be rescued by both PARP inhibitor and NAD^+^ precursors.^349^ Aging-related nuclear NAD^+^ decline inhibits the mitochondrially encoded genes via the SIRT1-HIF-1α-c-Myc pathway, while boosting NAD^+^ levels rescues the mitochondrial function in old mice in a SIRT1-dependent manner.^345^ NAD^+^ also affects the acetylation and activity of oxidative enzymes in mitochondria via altering SIRT3 activity. The circadian activity of SIRT3 induced by NAD^+^ oscillation regulates the rhythmic acetylation and activation of oxidative enzymes and respiration in isolated mitochondria.^350^

#### NAD^+^ ameliorates the oxidative damage during aging

**Fig. 6:**
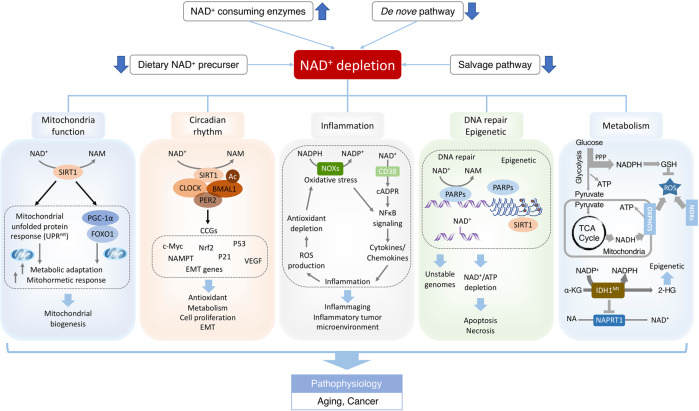
NAD^+^ deficits in aging-associated dysfunction and cancer. Environmental stimuli, including nutrient perturbation, infection, radiation and inflammation, induce oxidative stress, which causes the damage of cellular biomolecules and organelles. NAD^+^ and its metabolites function as crucial regulators to maintain cellular redox homeostasis through replenishing the reducing power or modulating the activity of NAD^+^-consuming enzymes including sirtuins and PARPs. However, disequilibrium of NAD^+^ metabolism could disturb physiological processes, including mitochondria function, circadian rhythm, inflammation, DNA repair and metabolism, leading to aging-associated dysfunction and cancer. NAD^+^ levels could be augmented by dietary NAD^+^ precursor, inhibitors of NAD^+^-consuming enzymes, caloric restriction and exercise. NAD^+^ boosters restore the bioenergetics, redox balance and signaling pathways, ameliorating the adverse effects of pathophysiological conditions, including infection, aging and cancer. Abbreviations: 2-HG, 2-hydroxyglutarate; α-KG, α-ketoglutarate; CCGs, clock-controlled genes; FOXO1, Forkhead Box O1; GSH, Glutathione; IDH1^Mt^, mutant isocitrate dehydrogenase 1; NOXs, NADPH oxidase; PER2, period circadian clock 2; PPP, pentose phosphate pathway; PGC-1α, peroxisome proliferator-activated receptor-gamma coactivator alpha; ROS, reactive oxygen species; OXPHOS, Oxidative phosphorylation; TCA cycle, tricarboxylic acid cycle

There is a growing awareness that oxidative damage is an essential driver of age-related deterioration in cell function.^351,352^ The DNA oxidative damage and protein oxidation in the aged human brain are associated with declined antioxidant enzyme activities.^353,354^ Age-related increase in oxidative stress and cell senescence leads cells/tissues to be more prone to undergo necroptosis, thereby releasing DAMPs that trigger the chronic inflammation observed with aging.^355^ The pro-inflammatory cytokines, in turn, augment both mitochondrial and NOX-generated ROS, contributing to further accumulation of oxidative damage (Fig. 6).^356–359^

NADH/NADPH is a powerful reduce source for buffering oxidative stress, thereby protecting cells/tissues from oxidative stress during aging. The remarkable reduction of NAD^+^ concentration and NAD^+^/NADH ratio in aged rats occurs in parallel with enhanced oxidative stress and diminished antioxidant capacity.^334^ NMN addition in isolated aortas elevates the NAD^+^ and MnSOD levels, thus enhancing the antioxidant capacity.^344^ The overexpression of Nmnat3 efficiently boosts NAD^+^ in a variety of murine tissues, which significantly suppresses the ROS generation, and protects from aging-related insulin resistance.^360^ Overexpression of G6PD promotes NADPH production, preventing tissue from oxidative damage to improve mice health span.^361^ NAD^+^ also regulates oxidative stress in cellular senescence by regulating sirtuins and PARPs. NAD^+^-dependent SIRT1 is significantly upregulated in response to oxidative stress, protecting heart from oxidative damage, contributing to retard of aging.^362^

#### NAD^+^ deficiency correlates with disturbed *circadian clocks* during aging

Besides mitigating the oxidative damage, NAD^+^ can extend lifespan by driving the circadian rhythms. The misalignment of the circadian clocks, the internal timekeeper mechanism that links metabolism with the exogenous and endogenous factors, has been associated with the acceleration of aging.^240^ The circadian sirtuins link the NAD^+^ metabolism to the circadian clock machinery during aging. SIRT1 induces the circadian transcription of core clock genes, such as Cry1, Per2, Rorγ, and Bmal1 via either rhythmically deacetylating BMAL1 or PER2.^36,38^ SIRT1 also modulates CLOCK-mediated chromatin remodeling at H3 Lys9/Lys14 at circadian promoters to control circadian.^38^ In the SCN of aged mice, SIRT1 level is significantly decreased, resulting in a reduction of BMAL1 and other circadian proteins.^363^ The autonomous hepatic clock induces the NAD^+^ salvage pathway to partially restore NAD^+^ oscillation, driving the SIRT1 circadian function in the liver even without inputs from other clocks.^241^ Therefore, NAD^+^-dependent SIRT1 regulates the aging-dependent decline in central circadian function.

### The critical role of NAD^+^ pool in tumorigenesis

NAD^+^ not only acts as a co-enzyme for metabolic redox reactions, but also functions as a co-substrate to modulate the activity of NAD^+^-consuming enzymes that govern the critical steps in tumorigenesis, including genome stability, metabolism, cell growth, cell death, redox homeostasis and immune response. The sirtuins and PARPs in tumorigenesis exhibit both oncogenic and tumor suppressor activity, which might be determined by their sublocalization and cell type (Fig. 6).

#### NAD^+^-related metabolic reprogramming and redox homeostasis in tumorigenesis

Cancer cells undergo metabolic reprogramming that provides the substrates and energy for biomass generation, to sustain the stress response and continuous proliferation.^364^ The metabolic reprogramming is characterized by shifting glucose metabolism to aerobic glycolysis, including enhanced cytosolic lactate fermentation and PPP and decreased OXPHOS. This shift not only allows for rapid production of energy but also for maintenance of NADH/NAD^+^ redox ratio, which is required for metabolic processes, such as aerobic glycolysis, the TCA cycle, OXPHOS, FAO, serine biosynthesis and antioxidant defense.^365–367^ Cytosolic NAD^+^ is required for glycolysis, in which GAPDH converts NAD^+^ to NADH. In health cells, cytosolic NADH is shuttled into mitochondria, where it is turned into NAD^+^ by OXPHOS whereas in cancer cells, the conversion of NAD^+^ to NADH in mitochondria is not sufficient for the high rate of glycolysis due to reduced OXPHOS. Therefore, cancer cells enhance the cytosolic lactate fermentation to generate NADH by LDHA. Activation of LDHA by oncogenic receptor tyrosine kinase FGFR1 promotes glycolysis and tumor growth by increasing the NAD^+^/NADH ratio,^368^ while the aberration of NAD^+^/NADH due to reduced activity of mitochondrial complex I promotes the aggressiveness of human breast cancer cells.^369^

The ‘hyper-metabolism’ of cancer cells causes the excessive generation of ROS.^370^ ROS contribute to tumorigenesis through multiple processes, including causing oxidative DNA damage, genomic instability and inflammatory stress to drive malignant transformation, and acting as a messenger to regulate signaling pathways to support tumor initiation, development, and angiogenesis.^142,261,272,371–374^ Cancer cells build a complicate and powerful antioxidant system, such as the GSH and Trx systems, to adapt to the high ROS levels. Notably, both GSH and Trx systems rely on the reducing power of NADPH, which is generated by G6PD in PPP, ME1 in glutamine metabolism and NNT. In cancer cells, increased ROS will oxidize the specific isoform of pyruvate kinase (PKM2), diverting glucose flux towards the PPP and generation of NADPH for GSH recycling.^96,375,376^ Similarly, nutrition stress or oxidative stress induces the expression of enzymes, including NAMPT, ME1 and NNT, which augments the NADPH to support the cell survival under glucose deprival and anoikis conditions, thereby promoting tumor growth and metastasis.^377–379^ IDH1 mutations in human cancers favor consuming the NADPH for 2-HG synthesis at the expense of other NADPH-requiring pathways essential for cell viability even when NADPH is limiting.^96^ Additionally, AMPK activation by reduced ATP levels maintains NADPH through inhibiting NADPH consumption in fatty-acid synthesis and enhancing NADPH production from fatty-acid oxidation instead of PPP to inhibit cell death.^380^ However, NAD^+^ depletion exacerbates oxidative damage via reducing the antioxidant defense capacity, resulting in impaired cell proliferation and increased cell death.^365,366^

The NAD^+^/NADH ratio emerges as a fine-tuned signal to regulate redox status through sirtuins.^26^ Sirtuins manipulate the metabolism reprogramming via directedly altering the activity of metabolic enzymes by NAD^+^-dependent modification or changing their expression by regulating transcription factors.^217^ All the enzymes but PGI in the glycolysis and the TCA cycle can be acetylated under the control of sirtuins. GAPDH and PKM2 are two major enzymes in glycolysis that regulated by sirtuins. SIRT1 can bind and retain GAPDH in the cytosol, but SIRT5 actives GAPDH via demalonylation, thereby elevating glycolytic flux.^381^ Both SIRT2-catalyzed deacetylation and SIRT5-mediated desuccinylation of PKM2 reduce the activity of PKM2, preventing the carbon entry into the TCA cycle. In contrast, SIRT3 deacetylates and activates PDHA1 acetylation, linking glycolysis and OXPHOS.^382^ SIRT3 and SIRT5 stabilize and activate the enzymatic activity of SHMT2 by its deacetylase and desuccinylase activity, respectively, thereby promoting the serine catabolism to drive carcinogenesis.^383^ SIRT5-catalyzed demalonylation and inactivation of SDHA block the TCA cycle and induce succinate accumulation, promoting tumorigenesis and drug resistance.^383,384^ Additionally, sirtuins also regulate the expression of metabolic enzymes via transcription factors, such as HIF-1α. SIRT3 enhances the enzymatic activities of SOD2 and IDH2 to limit ROS levels, which repress the metabolism reprogramming in cancer cell via destabilizing HIF-1α, thereby repressing tumor growth.^142,222,225,385–387^ It has also been demonstrated that the enzymes in glycolysis (e.g., GLUT4, HK1, GSK3B, and GAPDH), the enzymes (e.g., PDPR PDHA1, and PDHX) in carbohydrate metabolism, and most enzymes in ETC and ATP synthesis are assigned as ADP-ribose amino acid acceptor.^388^ However, whether the ADP-ribosylation of metabolic enzymes contributes to the metabolism reprogramming in cancer cells requires further investigation.

#### NAD^+^-regulated genome stability and gene transcription in carcinogenesis

NAD^+^ metabolism is not only essential for metabolic and redox homeostasis, but also required for epigenetic reprogramming in tumorigenesis. Genomic instability and altered transcriptional pattern are well-known hallmarks of cancer.^364^ Sirtuins and PARPs control the genome stability and gene transcription by regulating the histone modification, DNA repair, as well as the recruitment and activation of transcription factors.^169,389^ SIRT1 is responsible for the histone acetylation patterns, including the H4K4ac, H3K9ac, H4K16ac and H1K26ac, associated with tight chromatin compaction. The activity of SIRT1 also modulates the formation of H3K9me3, H4K20me3 and H3K79me2 to regulate chromatin. For instance, SIRT1 alters the acetylation patterns of histones H3 and H4, including H3k4ac, H3k9ac and H4k16ac, to regulate the expression of cancer-related genes in breast cancer.^390^ Besides the impact on chromatin state, SIRT1 modulates the non-histone proteins to initiate DNA repair and gene transcription. SIRT1 induces the recruitment of DNA repair factors, including NBS1 and Rad51, in keratinocytes to maintain genome stability and gene transcription.^174^ Oxidative stress-induced SIRT1 can deacetylase hMOF to reduce the expression of DNA repair proteins, including RAD50, BRCA2 and FANCA in human colorectal cancer cells.^169^ hMOF also plays crucial roles in transcription activation by H4K16 acetylation in HeLa and glioma cancer cells.^391,392^ The contradictory role of SIRT1 in cancer is manifested as their overexpression in neoplasms, such as prostate, AML, non-melanoma or melanoma skin cancer, and colon carcinomas, and the reduced expression in other cancers, including breast cancer and hepatic cell carcinomas.^393,394^ Thus, the mechanisms underlying the regulatory role of SIRT1 in DNA repair and gene transcription in cancer development need further exploration.

As aforementioned, PARPs govern the genome stability and gene transcription via NAD^+^-dependent ADP-ribosylation. PARPs-induced ADP-ribose marks elevate 10- to 27-fold in response to the oxidative genome damage by H_2_O_2_ in human osteosarcoma cells.^133,183,184^ A variety of cancers have somatic mutations resulting in genomic disability and defective DNA repair, including BRCA1/2, ATM, CHK2 and TP53.^395^ The loss of double-strand repair pathway due to BRCA1 or BRCA2 mutations renders cancer cells more dependent on the PARPs-mediated repair, and more sensitive to PARP inhibition, raising a possibility of a wider application of PARPi in cancer therapy.^396–398^

NAD^+^ metabolism is also linked to epigenetic modification by NNMT that transfers the methyl group of SAM to NAM. NNMT is increased in a broad range of cancers, such as papillary thyroid cancer, renal clear cell carcinoma, glioblastoma tumors, bladder cancer, colorectal cancers, gastric cancers, and oral squamous cell carcinoma.^200,399–402^ Elevated NNMT inhibits the methylation potential of cancer cells through inducing methyl sink in the form of MNA. Reducing the NNMT expression impairs the cell proliferation and tumor growth of mesenchymal glioblastoma stem cells (GSCs), accompanied by reduced methylation ability.^403^ Besides, NNMT promotes HCC cell invasion and metastasis by changing the H3K27me patterns and transcriptionally activating CD44. NNMT-mediated CD44 mRNA m6A methylation produces a CD44v3 splice variant, while MNA stabilizes CD44 protein by inhibiting the ubiquitin-mediated degradation.^404^ Furthermore, NNMT depletion elevates the NAD(H)^+^ levels that result in an enhanced expression of sirtuin target genes and a reduced H3K9Ac.^405^ Therefore, NNMT acts as a crucial metabolic modulator of epigenetic modification, promoting the migration, invasion, proliferation and survival of cancer cells.

The oncometabolite, 2-hydroxyglutarate (2-HG), also couples the NADP^+^/NADPH to epigenetic modification, including histone and DNA demethylases, in tumorigenesis. Mutant IDHs, accounting for 80% of lower-grade gliomas and secondary GBM, continuously produce 2-HG. To support 2-HG synthesis, cancer cells with IDH1^R132H^ mutation enhance NADPH production via the PPP.^406,407^ Interestingly, IDH1 mutants compete for NADPH to synthesize 2-HG with other pathways that are critical for cell viability, resulting in further disruption of cellular metabolism and redox homeostasis in tumorigenesis.^36^

#### NAD^+^-dependent cancer cell proliferation and metastasis

Given the massive demand for NAD^+^ to support the metabolism reprogramming, the genome integrity and gene transcription in tumorigenesis, cancer cells enhance the capacity of NAD^+^ production through various pathways. It has been demonstrated that tumors that arise from cells with highly NAPRT expression will rely on NAD^+^ de novo synthesis for survival. While cancers derived from tissues with normal NAPRT levels are entirely dependent on the NAD^+^ salvage pathway for survival.^408^ Both the upregulated NAPRT in ovarian cancer and the high expression of NAMPT in glioblastoma, colorectal cancer tumors, and breast cancer, increase intracellular NAD^+^ levels, contributing to cancer cell metabolism and DNA repair process in tumors.^409^ Moreover, resistant CCRF-CEM cells with high QPRT activity exploit amino acid catabolism as a substitute pathway for NAD^+^ generation.^410^

High expression of NAMPT or NAPRT is associated with tumor progression, invasion, and drug resistance.^411–414^ This effect is mediated by NAD^+^-dependent PARPs and SIRT1.^415–417^ SIRT6 induces the NAMPT activity to increase NAD^+^ content, thereby preventing oxidative damage. Activation of the c-MYC-NAMPT-SIRT1 feedback loop may crucially contribute to the initiation and development of both routes to colorectal cancer.^413,417^ Declining NAD^+^ levels reduced the SIRT1-mediated inhibition of STAT3, which induces the secretion of IL-6 and TFG-β to sustain the signaling required for EMT.^418^ CD38 expression inversely correlates with prostate cancer progression due to its ability to lower intracellular NAD^+^, resulting in cell-cycle arrest, reduced glycolytic and mitochondrial metabolism, and impaired fatty acid and lipid synthesis.^419^ These findings demonstrated a pro-tumor activity of NAMPT, suggesting a promising therapeutic target for cancer treatment. NAMPT inhibitors, FK866, STF-118804 and KPT-9274, can reduce the viability and growth of different cancer cells and have an additive effect in combination with main current chemotherapeutic drugs.^420–423^

### NAD^+^ metabolism and metabolic diseases

#### Diabetes

**Fig. 7:**
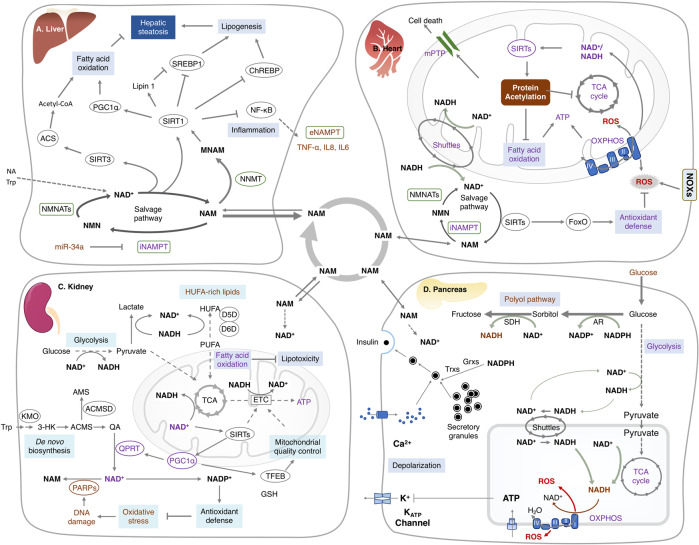
Pathophysiological role of NAD^+^ disarrangement in metabolic diseases. **a** The liver is a master organ of NAD^+^ metabolism and may facilitate the NAD^+^ biosynthesis in other tissues. NAD^+^ metabolism plays a critical role in the lipid metabolism through modulating the activity of sirtuins. The reduced NAMPT expression and NAD^+^ levels contribute to the development of NAFLD through manipulating dysmetabolic imbalance, hepatic energy homeostasis, glucose homeostasis, hepatic inflammation and insulin resistance. **b** Decreased NAD^+^/NADH ratio by the mismatch between NADH production and oxidation inhibits the activity of sirtuins in the failing heart. Elevated protein acetylation weakens the energy metabolism through negative feedback to OXPHOS and substrate metabolism, impairing antioxidant defense and sensitizing the mPTP to ROS or calcium. **c** The deduced NAD^+^ levels in kidney are attributed to the decreased expression of enzymes in NAD^+^ de novo synthesis and increased consumption by DNA damage activated PARPs. NAD^+^ depletion inhibits the SIRT1/PGC1α mediated mitochondrial quality control, ATP production and NAD^+^ de novo biosynthesis. The phosphorylation of NAD^+^ to NADP^+^ enhances the antioxidant defense against oxidant stress. NAD^+^-dependent defect in FAO results in intracellular lipid accumulation. In addition, the defected FAO and increased desaturation of PUFAs to HUFAs due to NAD^+^ deficiency and impaired mitochondrial function result in the accumulation of HUFA-containing triglycerides and cellular lipid in renal tubular cells. **d** The insulin secretion is adjusted by the dynamic glucose concentration in blood. As a master regulator of insulin secretion, glucose is metabolized via the glycolysis and TCA cycle to produce NADH and ATP. The increased NADH and ATP induces the closure of ATP-dependent K^+^ channels, the opening of voltage-gated L-type Ca^2+^ channels, the raising of cytosolic Ca^2+^ and culminating in insulin secretion in pancreatic β-cells. The activity of mitochondrial shuttles including the glycerophosphate and malate/aspartate shuttles allows the reoxidation of cytosolic NADH into NAD^+^, which is required for maintenance of the glycolysis. Purple representants the downregulated proteins or activated biological functions, while brown labels the upregulated proteins and repressed physiological activities. Abbreviations: ACMSD, alpha-amino-beta-carboxy-muconate-semialdehyde decarboxylase; AR, Aldose reductase; ETC, electron transport chain; Grxs, glutaredoxins; HUFAs, highly unsaturated fatty acids; KMO, kynurenine 3-monooxygenase; FAO, fatty acid oxidation; PUFAs, polyunsaturated fatty acids; SDH, Sorbitol dehydrogenase; Trxs, thioredoxins. 3-HK, 3-hydroxykynurenine

Diabetes is a chronic metabolic disease characterized by hyperglycemia. The human pancreas cannot produce enough insulin, or the body cannot effectively use the produced insulin, which causing a pathological increase in blood sugar. Pancreatic β-cells maintain systemic glucose homeostasis by controlling the release of insulin, thereby responding to changes in metabolic demand. The high capacity, low-affinity GLUT2 and high K_M_ glucokinase (GK) in β-cell ensure the proximal glucose-sensing. The glucose fluxes through the glycolytic pathway and the TCA cycle, enable the production of NADH and ATP. Thus, the elevated blood glucose levels lead to more production of NADH and ATP, resulting in closure of ATP-sensitive potassium channels, cell depolarization, Ca^2+^ influx and culminating in insulin secretion.^424–427^ In addition to NADH produced in the mitochondria by the TCA cycle, cytoplasmic NAD^+^ generation is essential for insulin secretion.^82,88^ Given that β-cells only have extremely low LDHA activity to regenerate NAD^+^ for glycolysis, NADH generated by glycolysis must be transferred into the mitochondria to be oxidized by complex I.^424^ Cytoplasmic NADH from the glycolytic pathway is delivered to mitochondria through two NADH shuttles, G3P shuttle and MA shuttle, allowing NAD^+^ recycling to sustain glycolytic flux. As evidenced, at maximal glucose stimulation, the rising of NAD(P)H levels is estimated to be approximately 30 mM in whole pancreatic islet beta cells, with a 7 mM in the cytoplasmic domain and an approximately 20 second delayed 60 mM in mitochondrial domain.^428^ The NADH shuttle thus promotes the increase of Ca2^+^ after the formation of mitochondrial membrane potential and sufficient ATP generation from ETC, concomitantly triggering glucose-stimulated insulin secretion (GSIS) (Fig. 7).^429–431^

Sustained high levels of insulin demand will eventually give rise to functionally compromised or physical loss of β-cells, which culminate in hyperglycemia and diabetes.^432–434^ However, β-cells exposed to diabetes and hyperglycemia exhibit striking changes in metabolism.^433,435^ Importantly, the increase of Krebs’ cycle in the mitochondrial that generally responds to glucose is terminated, which will cause glucose to fail to escalate the NADH and ATP content in the pancreatic islets of diabetic patients.^433^ Rather than producing from mitochondrial TCA cycle under controlled conditions, the NADH in diabetic islets is generated by cytoplasmic sorbitol oxidation and mitochondrial pyruvate oxidation in response to diabetes and hyperglycemia stimulate.^433,436^ In diabetes, when GPDH is inhibited due to the reduced utilization of NAD^+^, about 30% of glucose is involved in the polyol pathway. When β cells are over-nutrient and hyperglycemic, the excessive NADH produced by the polyol pathway will promote the production of superoxide through the overload complex I of ETC, resulting in cell dysfunction and impaired insulin secretion.^424^ Moreover, the two components of the G3P shuttle (including GPD1 in the cytoplasm and GPD2 in the mitochondria) are up-regulated in mRNA and protein levels in diabetic islets, thus ensuring the transfer of electrons from NADH produced in glycolysis to mitochondria.^429,433^ In line with that, the overexpression of cytoplasmic malic enzyme (ME1) enhanced the GSIS and anaplerosis in insulinoma cells.^437^ Selective reduction of cytosolic ME1 expression and enzyme activity significantly reduces GSIS and amino acid-stimulated insulin secretion (AASIS).^438^ There is growing evidence indicating that cytosolic NADPH is one of the effectory metabolic coupling factors, a variety of critical intermediates and cofactors involved in the GSIS. Although glucose causes a dose-dependent inhibition of pentose phosphate pathway activity in beta cells, the major pathway for NADPH production, the cytosolic ratio of NADPH/NADP^+^ increases during glucose-stimulated insulin release.^439–441^ NADPH stimulates the exocytotic machinery by the redox proteins glutaredoxin and thioredoxin and has a local redox reaction, thereby accelerating exocytosis and promoting the secretion of insulin in pancreatic β-cells.^442,443^

The NAD^+^ level is associated with insulin resistance. HFD significantly impairs the role of NAMPT-regulated NAD^+^ biosynthesis in metabolic organs.^337^ Mice that specifically knock out the *Nampt* gene of adipocytes have serious insulin resistance, which is manifested by an increase in plasma free fatty acid content and a decrease in plasma content of the main insulin-sensitive adipokine, adiponectin. These deleterious alterations can be normalized by administering NMN.^444^ Furthermore, NMN alienates glucose intolerance and lipid profiles by recovering NAD^+^ levels in age induced T2D mouse model.^337^ Conversely, overexpression of *Nmnat3* in mouse can effectively increase NAD^+^ levels in a variety of tissues and prevent aging-related insulin resistance caused by diet.^360^ Owing to its expression and activity increase with age, CD38 is essential for age-associated NAD^+^ decrease through degradation of NMN in vivo. CD38 deficiency has improved glucose intolerance with HFD, which could be further ameliorated by supplement of NR.^52^ 78c, as a highly specific and effective CD38 inhibitor, can reverse age-associated NAD^+^ reduction and improve some metabolic and physiological parameters of aging, such as glucose tolerance, cardiac function, muscle function and exercise capacity in both natural aging and accelerated aging mice models.^53^

#### Obesity

The pathological expansion of adipose tissue is specifically manifested in the dysregulated production of adipokines and lipid, low-grade inflammation and enrichment of extracellular matrix. Insulin resistance is a critical whole-body abnormal metabolism closely related to obesity.^445,446^ A reduction of NAD^+^ levels in cells is observed in many tissues with obesity, like the skeletal muscles, hypothalamus, liver and adipose tissue.^337,447^ Supplementation of NR or NMN can protect against the decrease of NAD^+^ levels, and partially inhibit the weight gain of the mice fed with HFD by enhancing energy expenditure.^448^ The NAD^+^ biosynthesis regulated by NAMPT in adipocytes plays an important role in the pathogenesis of obesity-related metabolic complications.^444^ Both the expression of NAMPT in visceral fat and the level of NAMPT in serum are positively correlated with the degree of obesity.^449–453^ In contrast, obese subjects have lower levels of NAMPT in subcutaneous fat tissue.^454–456^ The upregulation of NAMPT by the activation of the HIF1-α pathway under hypoxic conditions plays a vital role in processing dietary lipids to regulate the plasticity of adipose tissue, whole-body glucose homeostasis and food intake. The deficiency of adipose *Nampt* can partially reduce food intake, thereby preventing obesity caused by diet. In addition, NAMPT-mediated NAD^+^ biosynthesis plays a vital role in adipose by promoting weight gain caused by HFD, which can be proved by the inability of HFD-fed FANKO mice that can expand adipose tissue normally.^453,457^

Several studies have shown that in different adipocyte models, such as primary adipocytes, 3T3-L1 preadipocyte cell and SGBS cell, NAMPT can be secreted directly into the supernatant through non-classical pathways. These results indicate that the adipose tissue is one of the main sources of secreted extracellular NAMPT (eNAMPT).^458^ Treatment with eNAMPT can increase the expression of lipoprotein lipase and PPARγ in preadipocytes and promote the expression of fatty acid synthase in differentiated adipocytes, which indicates that eNAMPT may be a positive regulator in adipocytes lipid metabolism.^459^ Adipose tissue-specific *Nampt* knockin and knockout mice (ANKI and ANKO) showed opposite alterations of circulating eNAMPT, which accordingly affected hypothalamic NAD^+^/SIRT1 signaling and physical activity. Treatment with NMN can improve physical activity deficits in ANKO mice.^460^ The biosynthesis of NAD^+^ in adipocytes is crucial for the extension of HDF-induced white fat depots and may have more specific effects in lipid accumulation and processing.^457^ Based on these observations, the effect of NAMPT on obesity depends on its enzymatic activity. Increasing NAD^+^ levels by supplementing NR in mouse tissues and mammalian cells activates SIRT1 and SIRT3, which ultimately leads to increased oxidative metabolism and prevents metabolic abnormalities induced by HFD.^37^ Moreover, adding leucine to HFD can increase the expression of NAMPT and SIRT1 and elevate the level of NAD^+^ in cells, which will reduce the acetylation of FoxO1 and PPAR-γ co-activator 1α (PGC1α). Deacetylation of PGC1α may promote the upregulation of genes related to fatty acid oxidation and biogenesis in mitochondria, thereby restoring mitochondrial function and protecting against HFD-induced obesity.^461^

#### Non-alcoholic fatty liver disease (NAFLD)

Alterations of hepatic metabolism are critical to the development of liver diseases, in which the NAFLD is the most common chronic liver disease and is strongly related to metabolic syndrome. NAFLD might eventually cause more severe liver diseases, such as liver fibrosis, liver cirrhosis, liver failure and hepatocellular carcinoma (HCC).^462–464^ It is reported that reduced NAD^+^ concentration caused a dysmetabolic imbalance, leading to the development of NAFLD.^465^ Oral administration of NR halts the progression of NAFLD through rescuing the NAD^+^ reduction, reducing the total cholesterol and triglyceride levels, decreasing the AST level, and reversing Kupffer cells accumulated and inflammatory in aged group.^466,467^ Troxerutin, as a derivative from natural bioflavonoid rutin, could promote NAMPT expression to restore the NAD^+^ level depleted by oxidative stress in the HFD-induced NAFLD mouse model (Fig. 7).^468^ In a transgenic mouse model of DN-NAMPT, the researchers found that middle-aged mice had a systemic reduction of NAD^+^ and showed a moderate NAFLD phenotype, like triggered inflammation, lipid accumulation, increased oxidative stress and impaired insulin sensitivity of liver. Some of these phenotypes are further exacerbated after feeding a high-fat diet. However, oral NR can completely reverse these phenotypes caused by NAD^+^ deficiency or high-fat diet.^335^ Significantly, knockdown of Nampt gene increases, while over-expression reduces hepatic triglyceride both in vitro and in vivo models. The expression of NAMPT in patients with NAFLD has decreased systemically both in serum and within the hepatic tissue, which is regulated by PPARα activation and glucose.^469^ Meanwhile, the NAMPT is also a target of FoxO transcription factors that control the NAD^+^ signaling in the regulation of hepatic triglyceride homeostasis.^470^ Additionally, the hepatic microRNA-34a, which is increased in obesity, reduces NAD^+^ levels and SIRT1 activity by targeting NAMPT. The antagonism of microRNA-34a could moderate inflammation, steatosis and glucose intolerance, and recover NAMPT/NAD^+^ levels in diet-induced obese mouse model.^447^ It was found that higher level of NAMPT in serum in women is correlated with a much lower hepatic de novo lipogenesis (DNL) index, although they do not correlate with the DNL index, but had a correlation with a higher hepatic fat in men, implying a sex-dependent interpretation role of serum NAMPT level for NAFLD prognosis.^471^ Mechanistically, reduced NAMPT/NAD^+^ inhibits SIRT1’s function, thereby attenuating the deacetylation activity of SREBP1, leading to the expression of ACC and FASN.^463^ Conversely, stabilization of SIRT1 by increasing NNMT expression or MNAM levels improves lipid parameters.^63,472,473^

Beyond the metabolic activity, NAMPT may also participate in NAFLD’s pathogenesis by controlling hepatic inflammation, insulin resistance and glucose homeostasis.^474,475^ eNAMPT regulates glucose production via the PKA/CREB signaling in HepG2 cells.^476^ The expression of NAMPT is closely related to the expression of pro-inflammatory cytokines in the inflammation induced by free fatty acids and is remarkably reduced by the inhibition of NF-κB in HepG2 cells.^477^ To date, the clinical analysis reveals controversy regarding the relationship of circulating NAMPT with NAFLD. Several studies report no statistically significant difference in NAMPT levels between NAFLD and healthy controls, as well as among different histological NAFLD groups.^478,479^ Another study shows that NAFLD patients have systemically decreased the expression of NAMPT in both serum and hepatic tissue.^469^ In contrast, the liver and serum NAMPT of morbidly obese women with NAFLD are significantly higher than that of morbidly obese women with healthy livers.^480^ The serum NAMPT level and its expression in hepatic tissues are positively correlated with pro-inflammatory factors.^480^ Moreover, the expression of NAMPT is notably higher in fibrosis patients and is correlated positively with the stage of fibrosis in NAFLD patients.^481^ Elevated serum NAMPT, together with inflammatory factors, such as IL-6, IL-8 and TNF-a, is associated with an increased likelihood of exhibiting NAFLD and NASH.^482^ Given that hepatocytes are only one of the sources of eNAMPT, circulating NAMPT levels may not represent its actually concentration in local liver or adipose tissues, thus requiring further research to determine its exact role in NAFLD.

### NAD^+^ metabolism in neurodegenerative disorders

**Fig. 8:**
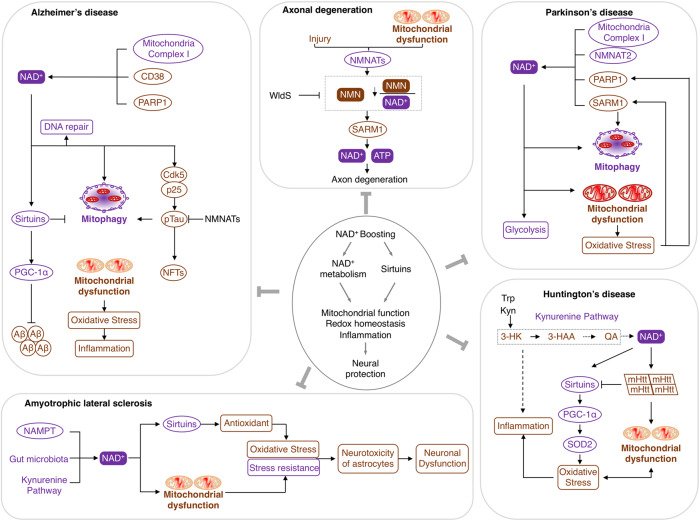
Linkages between NAD^+^ depletion and neurodegenerative disorders. Most neurodegenerative disorders, including axonal degeneration, Alzheimer’s disease (AD), Parkinson’s disease (PD), Huntington’s disease (PD) and Amyotrophic lateral sclerosis (ALS), are associated with mitochondrial dysfunction, lowered antioxidant capacity and heightened mitophagy, all of which are converged into the age-related NAD^+^ depletion induced by either enhanced consumption or impaired biosynthesis. These neural pathologies can be rescued by NAD^+^ boosting. Purple representants the downregulated proteins or activated biological functions, while brown labels the upregulated proteins and repressed physiological activities in neurodegenerative disorders. Abbreviations: mHtt, mutant Huntingtin; Aβ, amyloid beta; NFTs, neurofibrillary tangles; 3-HAA, 3-hydroxyanthranilic acid; QA, quinolinic acid; WldS, slow Wallerian degeneration; 3-HK, 3-hydroxykynurenine

Neurodegenerative diseases are a heterogeneous group of diseases, including Alzheimer’s disease (AD), Parkinson’s disease (PD) and amyotrophic lateral sclerosis (ALS), which are characterized by progressive degeneration of the structure and function of the peripheral and central nervous system, with the characteristics like the accumulation of misfolded and aggregated proteins that are associated with severe proteotoxic stress (Fig. 8).

#### Axonal degeneration

Axonal degeneration is an early and prominent feature of many neurological disorders, including AD, PD, ALS, ischemic brain and spinal cord injuries, diabetic neuropathy and traumatic brain injury.^483,484^

SARM1, as the evolutionary conservative executor of degradation cascade, is required for the progression of rapid Wallerian degradation. The TIR domain of SARM1 has inherent NADase activity, which can cleave NAD^+^ into nicotinamide, cADPR and ADPR. The nicotinamide acts as a feedback inhibitor of SARM1. Axons require the NADase activity of the full length SARM1 to facilitate axonal degeneration and NAD^+^ consumption after injury.^33,34,485^ Similarly, the loss of endogenous NMNAT2 is an important cause of axon degeneration after injury.^486^ The axon damages caused by SARM1 or NMNAT2 can be restored by increasing NAD ^+^ synthesis, like over-expressing the NMNAT2 enzyme.^34,487^ The naturally occurring mutant mice, Wallerian degeneration slow (Wld^S^) with chimeric gene containing the N-terminal 70AA of UBE4B and full length NMNAT1, show a delayed Wallerian degeneration phenotype.^488–492^

Several mechanisms are underlying the protective role of NMNAT on severed axons.^493^ Firstly, NMNAT acts as a stress-response protein that aids the clearance or refolding of misfolded proteins like a chaperone.^494,495^ Secondly, NMNAT and WldS proteins facilitate axon preservation by suppressing the accumulation of NMN. The activity of NMNAT1 is essential for axon survival because activity reduced mutants have no axon protection effect. The protection effect can also be abolished by the expression of exogenous NMN and ectopic expression of NMN deamidase.^489,496^ Thirdly, NMNAT1 does not change the NAD^+^ biosynthesis, but prevents the SARM1-dependent NAD^+^ depletion caused by injury, which is important for axon degeneration.^490^ Furthermore, *Sir2*, as a mammalian homolog of SIRT1, is the downstream effector of increased Nmnat activity, which can lead to axon protection in Wallerian degeneration slow mice.^491^ Additionally, SARM1 protein is required for NMN to promote axon degeneration and Ca^2+^ influx. SARM1 and NMN play a role in common signaling, which ultimately leads to the increase and breakage of Ca2^+^ in axons and the dissociation of mitochondrial dysfunction.^497^ Although the inhibitor of NMN synthase NAMPT reduces NAD^+^ level, it can still provide strong morphological and functional protection for damaged synapses and axons.^489^

#### Alzheimer’s disease (AD)

AD is a long-term chronic disease in the prodromal and preclinical stages with an average course of 8 to 10 years, which is the most common neurodegenerative disorder.

Currently, amyloid β peptide (Aβ), APOE and microtubule-associated protein tau are three important factors that have sufficient evidence as the etiology of AD.^498^ The key pathological features of AD are the accumulation of Aβ-enriched neuritic plaques and neurofibrillary tangles (NFTs) (consisting of tau protein).^499^ Aβ and phosphorylated Tau (p-Tau) neurofibrillary lesions lead to the pathology of neurons that display oxidative damage, impaired Ca2+ processing, reduced DNA repair, dysfunction of lysosomal and mitophagy, all of which have a positive correlation with the age-related NAD^+^ decline.^500–504^ Mounting evidence supports the promotion of NAD^+^ consumption on the process of AD, whereas the accumulation of NAD^+^ suppresses the AD-related pathological progress and the decline of cognitive function in different AD model ranging from *C. elegans* to mice.^187,500,505,506^ The increased activity and expression of CD38 following aging is responsible for the age-associated decrease in NAD^+^ level and defective mitochondrial function, which is at least partially regulated by the NAD^+^/SIRT3 signaling pathway.^52^ CD38 deficiency in APPswePS1DE9 mouse reduces soluble Aβ concentration and Aβ plaques, correlated with improved spatial cognition.^507^

The brains of individuals with preclinical AD (PCAD) and AD exhibit oxidative damage to a variety of molecules, e.g., accumulation of protein carbonyls (PCs) in regions that are rich in Aβ-peptide-containing SPs, increased lipid peroxidation in AD and PCAD hippocampi and elevated DNA damage.^508^ Recent studies have found that damaged cellular energy expenditure and DNA repair are related to the AD’s pathogenesis.

In the novel AD mice with DNA repair defects (3xTgAD /Polβ^+/-^), the content of NAD^+^ is reduced. Increasing NAD^+^ by supplementing NR can remarkably normalize DNA damage, p-Tau, synaptic transmission and neuroinflammation, improve the ability of motor function, memory and learning and increase the activity of SIRT3 in the brain.^187^ Notably, NAD^+^ augmentation improves DNA repair through improving the neuronal NHEJ activity in AD mice.^186,187,509,510^ The accumulated DNA oxidative damage in AD hyper-activates the DNA damage sensor PARP-1, thereby reducing the concentration of cellular NAD^+^ and suppressing the function of NAD^+^-SIRT1-PGC-1α axis, which in turn causes abnormal mitochondria.^511^ Replenishing cellular NAD^+^ can promote DNA repair in neurons and restore mitochondrial function through mitophagy.^511^ Mitophagy diminishes insoluble Aβ_1-42_, Aβ_1-40_, and hyper-phosphorylated tau, preventing cognitive or memory impairment in mouse model.^500,512^ Additionally, NAD^+^ protects neurons against p-Tau pathologies. NAD^+^ accumulation may inhibit the phosphorylation of different Tau protein sites by inhibiting the activity of Cdk5-p25 complex.^501^ Nicotinamide, as a competitive inhibitor of sirtuins, specifically reduces the phosphorylation of tau (Thr231), which is related to microtubule depolymerization in an analogous manner to that of SIRT1.^513^ NMNAT, as a binding partner of HSP90, can specifically recognize p-Tau to inhibit its amyloid aggregation in vitro and reduce its symptoms in the fly tauopathy model, and this effect could be competitively destroyed by its enzymatic substrate.^514^

#### Parkinson’s disease (PD)

PD is a common neurodegenerative disease, mainly including motor and non-motor symptoms.^515^ The neurons of PD patients exhibit symptoms, such as mitochondrial dysfunction, oxidative stress and NAD^+^ metabolic changes. Maintenance of NAD^+^ levels is vital for proficient neuronal function.^333^ It is reported that LRRK2 G2019S dopaminergic neurons exhibit a decreased NAD^+^ pool and a reduced sirtuin deacetylase activity, correlating with elevated acetylation of sirtuin substrates p53, α-tubulin and SOD2.^516^ In the primary cell (sPD cell) derived from patients with sporadic PD, the cytosolic conversion of pyruvate to lactic acid resulted in a significant increase in the nuclear NAD^+^ level and cellular NAD^+^/NADH ratio. The alteration of NAD^+^ metabolism in sPD cells contributes to the activation of SIRT2 and subsequently reduces the acetylation level of α-tubulin. Inhibition of the deacetylase function of sirtuin-2 can enhance the acetylation of α-tubulin and facilitate the clearance and transport of misfolded protein.^517^ The redox status of NAD^+^/NADH is remarkably decreased in iPSC neurons from GBA mutation associated PD, which may be due to the decrease of NMNAT2, as evidenced by the significant increase after NMN treatment.^518^ Furthermore, increasing the level of available NAD^+^ by supplementing diet containing NAD^+^ precursors or inhibiting the activity of NAD^+^-dependent enzyme (e.g., PARP that snatches NAD^+^ competing with mitochondria) is a feasible strategy to avoid the neurotoxicity related to mitochondrial dysfunction.^518–520^ Besides, through maintaining the rate of NAD^+^ production and normalizing the NADH/NAD^+^ balance, both pentylenetetrazole (PTZ) and niacin (NA) exhibit neuroprotective properties.^521,522^

SARM1 participates in the occurrence of PD mainly through its enzyme activity of NAD^+^ degradation. Compared with healthy people, the phosphorylation level of SARM1 is significantly increased in the neuronal cells from PD patients, with a high sensitivity to oxidative stress. In the case of oxidative stress, JNK increases the phosphorylation of SARM1, resulting in enhanced NAD^+^ degradation activity, which in turn promotes the suppression of mitochondrial respiration.^523^ The binding of SARM1 and PINK1 can facilitate TRAK6-induced ubiquitination of lysine 433 on PINK1, which is essential for keeping the stabilized status of PINK1 and bringing it into the outer membrane of mitochondria. Down-regulation of SARM1 and TRAF6 reduce the level of PINK1 and then recruits Parkin to the impaired mitochondria, indicating that SARM1 plays a crucial role in mitophagy via modulating PINK1.^524^

There is evidence showing that supplementation of high-dose NAD^+^ precursors in cells or Drosophila can alleviate the pathological phenotype by reducing oxidative stress and mitochondrial damage and improving motor function, which provides a feasible solution for the prevention and improvement of PD.^525,526^

#### Huntington’s disease (HD)

HD, also known as Huntington’s chorea (Huntington’s chorea), is an autosomal dominant hereditary neurodegenerative disorder that has a tremendous devastating effect on patients and their families, characterized by chorea-like movements, cognitive decline and psychotic-like symptoms. It is caused by repeated amplification of the CAG trinucleotide in the huntingtin (Htt) gene on the short arm of chromosome 4, which leads to a prolonged polyglutamine stretch at the N-terminus of the protein.^527–530^ Mitochondrial defection and increased oxidative stresses are the most prominent features in the cells of HD patients.

SIRT3, as a deacetylase in mitochondria, modulates the transcription of mitochondrial in response to oxidative stress.^531^ Notably, the expression of the Htt mutant reduces the deacetylase effect of SIRT3. In the HD model, reduced expression and deacetylase activity of SIRT3, which in turn prevents the deacetylation of LKB1 and SOD2, leading to a decrease in the level of NAD^+^, defects in mitochondrial biogenesis and accumulation of ROS.^532^ However, the overexpression of SIRT1 displays protection against abnormal motor function, cortical and striatal atrophy and loss of striatal neurons in the transgenic HD mice via regulating mitochondrial function.^531^ Both SIRT1 and SIRT3 exert their function through PGC-1α, which acts as a modifier in HD and ALS patients and other models. SIRT1/3-PGC-1α pathway in HD transgenic mice attenuates motor deficits and neurodegeneration by alleviating oxidative stress, eliminating huntingtin aggregates and restoring mitochondrial function.^531,533–537^

It has been reported that the kynurenine pathway (KP) is closely related to the pathogenesis of HD. The degradation process of tryptophan in KP produces a variety of neuroactive metabolites with amino acid-like structures, such as an N-methyl-D-aspartate (NMDA) receptor agonist quinolinic acid (QA), and a neuroprotective NMDA receptor agonist kynurenic acid (KYNA).^538,539^ Compared with the control group, the ratio of kynurenine (KYN) to KYNA in HD increases significantly, which is related to the decrease in the production of KYNA in HD patients.^539^ Genetic and pharmacological inhibition of TDO and KMO can increase KYNA, but not the level of neurotoxic product 3-HK, thereby improving the symptoms of neurodegeneration.^538^

#### Amyotrophic lateral sclerosis (ALS)

ALS is a neurological disorder that causes progressive degeneration of the motor neurons in the brainstem, spinal cord and cerebral cortex.^540,541^ More than 25 gene mutations have been reported to be closely related to ALS, of which C9orf72 repetitive amplification mutations and SOD1 mutations are the most common causes. Among them, ALS caused by mutations in the antioxidant enzyme SOD1 accounts for about 1–2% of sporadic ALS (SALS) and 20% of familial ALS (FALS).^542–547^ Astrocytes are specific contributors to spinal motor neuron degeneration in SOD1-related ALS.^548^ The spinal cord astrocytes isolated from SOD1G93A transgenic rats were co-cultured with motor neurons, resulting in the induction of motor neuron death.^549^ The reactive astrocytes can promote excitotoxic injury of motor neurons by producing nitric oxide and peroxynitrite, which cause mitochondrial damage and apoptosis in cultured neurons, decreasing glutamate transport, releasing pro-apoptotic mediators selectively toxic to motor neurons.^550^ Enhancing NAD^+^ availability can abrogate the neurotoxicity of astrocytes from diverse ALS models. Either overexpression of NAMPT or supplementation of NMN can increase the mitochondria and total cellular NAD^+^ levels of ALS astrocytes, thereby enhancing the ability to resist oxidative stress and restoring the toxicity of astrocytes to motor neurons.^551^ The NR repletion increases the levels of UPRmt-related proteins and promotes the clearance of mutant hSOD1 neurotoxic protein.^552^ Moreover, supplementation of NR can reduce the expression of neuroinflammation biomarkers in the spinal cord, alleviate the degeneration of motor neurons and appropriately extend the survival time of hSOD1G93A transgenic mice.^540,541^

Studies have found that ALS is associated with NAD^+^ metabolism through KP pathway damage. The impairment of KP in ALS is confirmed by the following evidence: higher serum tryptophan, cerebrospinal fluid (CSF), KYN and QA and a decrease in serum picolinic acid levels in ALS patients. Moreover, both the inflammation of microglia in the motor cortex and spinal cord of ALS patients and the expression of IDO and QA in neuronal cells and microglia increased significantly.^501,553^ In parallel with the impaired de novo pathway, the NAD^+^ decline in the ALS might also be due to the inadequate NAMPT-mediated NAD^+^ salvage synthesis pathway. NAM, a metabolite in the salvage pathway, is reduced in both CSF and serum from ALS patients compared with healthy people. NR supplement can increase the NAD^+^ concentration, dependent on NRKs (NR kinases), thereby avoiding the need for NAMPT in the salvage synthesis pathway.^552,554^

The protective effect of NAD^+^ on ALS might also be linked to the altered activities of SIRTs, however, the conclusions of many studies are quite different. It has been found that the expression level and function of Sirt3 is selectively reduced in the spinal cord of SOD1G93A mice at the end of ALS course, while the level of Sirt3 is increased in the human spinal cord after autopsy.^555^

The overexpression of NAD^+^ dependent deacetylase SIRT6 and SIRT3 can eliminate the neurotoxicity in the astrocyte cultured in vitro by activating the Nrf2-mediated antioxidant defenses.^556^ Interestingly, the expression of SIRT1 in the spinal cord of WT mouse is reduced during normal aging, while the expression of SIRT1 in different regions of the brain (including the spinal cord, hippocampus, thalamus and cerebral cortex) in SOD1G93A mouse is increased.^557^ Besides, overexpression of SIRT1 in motor neurons slows down the progression of ALS in severely phenotypic SOD1G93A (with high copy numbers) mice, partly by activating the HSF1/HSP70i molecular chaperone system.^558,559^ However, SIRT1 and SIRT2 are generally reduced in ALS primary motor cortex while they are upregulated in the spinal cord in human post-mortem tissues. In contrast to the neuroprotection role of SIRT1, SIRT2 upregulation is toxic to neuronal cells.^540,557,560–564^ A preliminary clinical trial has confirmed the importance of SIRT1 activation and NAD^+^ metabolism in ALS. The drug used in this trail is EH301 (a mixture of pterostilbene and NR), which contains the SIRT1 activator and the precursor of NAD^+^. Compared with the placebo control group, EH301 can significantly alleviate the development of ALS.^501,565^

### NAD^+^ in cardiac and renal diseases

#### NAD^+^ and heart failure (HF)

HF is a complex clinical syndrome caused by various initial heart damage and subsequent disturbance in compensatory effects and pathogenesis mechanisms. It is manifested through a number of complex molecular and systemic dysfunctions from the subcellular level to the multi-organ system of the whole body.^566,567^ There are many kinds of evidence indicating that the imbalance of myocardial NAD^+^ pool is causally linked to metabolic remodeling and mitochondrial dysfunction in HF.^568^ A wide range of tissues display a reduction of NAD^+^ in the aging mice model. The down-regulation of NAD^+^ levels in the heart is most significant. NAD^+^ is reduced by 70% within 3 to 24 months, and as a compensation, the concentration of NADH has increased by 50%.^334,569^ The increased NADH/NAD^+^ ratio and protein hyperacetylation are found in the HF patients’ hearts and pathologically hypertrophied mice.^570^ It is important to note that hyperacetylation of mitochondrial proteins is considered to be an inducer for cardiac dysfunction.^570–572^ Increasing NAD^+^ levels by activating the NAD^+^ salvage pathway can inhibit mitochondrial protein hyperacetylation and cardiac hypertrophy and improve cardiac function under stress. Proteomics analysis identified a subgroup of mitochondrial proteins, particularly sensitive to the changes of NADH/NAD^+^ ratio. It is reported that the hyperacetylation modification of mitochondrial proteins caused by the imbalance of NAD^+^ redox mainly promotes the pathological remodeling of the heart through two different mechanisms. First, the hyperacetylation of the mitochondrial malate-aspartate shuttle protein inhibits the oxidation and transport of NADH in the mitochondria, resulting in an imbalance of redox in the cytoplasm. Second, the acetylation modification of the oligomycin-sensitive conferring protein increases its binding to cyclophilin D and enhances the sensitivity of the mitochondrial permeability transition pore. Both of these conditions can be restored by regulating the normalization of NAD^+^ redox balance at the genetic or pharmacological level.^570^

It has been reported that hyperacetylation of cardiac protein in mice with HFD is closely related to the decreased expression of SIRT3.^573^ Exogenous supplementation of NAD^+^ can maintain the intracellular NAD^+^ level and block the symptoms of agonist-induced cardiac hypertrophy in vitro and in vivo by regulating the activation of SIRT3.^574^ As a negative regulator in cardiac hypertrophy, SIRT3 protects the heart by inhibiting intracellular ROS.^140^ Mice lacking SIRT3 become more sensitive to the stimulation of several pharmacological or non-pharmacological stressors, showing symptoms, such as fibrosis, cardiac hypertrophy and high mortality.^575^ In the SIRT3 knockout mouse model, the progression of fibrosis and cardiac hypertrophy also accelerated with age. In addition, mitochondrial swelling also appears to increase with age due to the increased opening of mitochondrial permeability transition pore (mPTP).^575^ Similarly, the loss of mitochondrial complex I leads to a decrease in the ratio of NAD^+^/NADH and can inhibit the activity of Sirt3, thereby enhancing the protein acetylation and mPTP sensitivity. Supplementing cKO mice with NAD^+^ precursors can partially normalize the NAD^+^/NADH ratio, acetylation of protein and sensitivity of mPTP.^576^ It is noteworthy that compared with normal mice, SIRT3-deficient mice can cause increased oxidation of fatty acid in heart, which is due to the high acetylation modification and high activity of β-HAD and LCAD.^573^ Moreover, in the absence of sirt5, the succinylation of protein lysine mainly occurs in the heart. When fasting, Sirt5 knockout mouse has reduced ECHA (an enzyme regulates the oxidation of fatty acid) activity, increased long-chain acyl-CoAs content and decreased ATP content.^229^ Compared with control mice in the same litter, after 20 min of ischemia and 90 min of reperfusion, the area of cardiac infarction in Sirt5 knockout mice was larger. The ischemia-reperfusion injury (I/R injury) of Sirt5 knockout mouse heart is restored to normal levels by dimethyl malonate (a succinate dehydrogenase (SDH) inhibitor) pretreatment, which implies that the change of SDH activity is the leading cause of the damage.^577^ Clinically, I/R injury occurs during myocardial infarction or blood supply stop during cardiovascular surgery. I/R injury is related to the decrease of endogenous NAMPT and the down-regulated expression of SIRT1, SIRT3 and SIRT4. NAMPT strictly controls the NAD^+^ and ATP content, thus playing an important part in regulating cell survival by suppressing apoptosis and increasing autophagy flux in cardiomyocyte.^578–580^ SIRT3 reduction can increase the sensitivity of heart-derived cells and the adult heart to I/R injury and may cause more severe I/R injury in the elderly heart.^581,582^ Exogenous expression of NMN can activate Sirt1-mediated FoxO1 deacetylation, which can protect the heart from I/R injury during ischemia and reperfusion. Similarly, calorie restriction promotes NAD^+^ to stimulate the Nampt-Sirt1 signaling pathway, which can protect the heart from I/R injury by up-regulating antioxidants and down-regulating pro-apoptotic molecules by activating FoxO.^579,580,583–585^

It is reported that the human cardiac fibroblasts with high expression of NOX5 and NOX4 are the main source of cardiac fibrosis related to heart failure and cardiac hypertrophy. NADPH produced by G6PD increases the level of NOXs, thereby producing most of the superoxide during the course of heart failure in patients with ischemic cardiomyopathy. Under the acceleration of TGF-β1, Nox4 mRNA is significantly upregulated and mediates the transformation of fibroblasts into myofibroblasts by activating TGF-β1-Smad2/3 signaling, which leads to cardiac fibrosis.^586,587^

#### NAD^+^ and kidney failure

Acute kidney injury (AKI) is a common clinical syndrome, and its prevalence and mortality increase with age. In the AKI mouse model, compared with the kidneys from 3-month-old mice, NAD^+^ levels in the kidneys from 20-month-old mice were significantly reduced.^588^ Renal ischemia impairs de novo NAD^+^ biosynthesis via reducing the renal expression of QPRT. Knockout of one allele of QPRT recapitulates these effects and increases susceptibility to AKI compared with control mice, which could be prevented by augmenting NAD^+^ metabolism with oral NAM supplementation.^589,590^ The robust finding that the early rise of urinary quinolinate levels and the urinary quinolinate/tryptophan ratio are related to the probability of AKI and adverse outcomes in a cohort of >300 patients indicates that impaired NAD^+^ metabolism leads to kidney injury in patients.^591^ Additionally, boosting NAD^+^ levels via inhibiting ACMSD (an enzyme restricts the de novo synthesis of NAD^+^ from tryptophan) also protects against AKI after renal I/R injury.^15^ The decrease in enzymes related to NAD^+^ de novo synthesis is due to the inhibition of the activity of PPARγ coactivator 1α (PGC-1α), which is a crucial determinant of renal recovery from AKI.^592,593^ Moreover, due to the decrease of 3-hydroxykynurenine (a cytotoxic metabolite of KMO), the mice that lack active kynurenine 3-monooxygenase (KMO) will not develop into AKI after I/R injury.^590^

The expression of PARPs in the injured kidney’s proximal tubules is upregulated from 6–12 h after I/R injury.^594^ Oxidative stress and DNA damage caused by I/R injury lead to excessive activation of PARPs.^595^ A study suggests that the activation of PARP may lead to cell death through ATP consumption and enhancement of the inflammatory cascade in mice.^596^ Inhibition of excessive activation of PARP can protect renal function from abnormal hemodynamics, renal metabolic disorders and renal cell apoptosis during AKI.^594,597^ Meanwhile, knocking out the Parp1 gene can protect mice from ischemic kidney damage.^596^

The simultaneous effects of impaired NAD^+^ biosynthesis and over depletion of NAD^+^ by PARPs can lead to a decrease in the level of NAD^+^ in the kidney during AKI. The decline in NAD^+^ levels therefore results in impaired metabolism and mitochondrial function via SIRTs and PGC-1α.^593,598,599^ SIRT3 protects against mitochondrial damage in the kidneys by attenuating oxidative stress, inhibiting inflammation and inducing autophagy through regulation of the AMPK/mTOR pathway.^600,601^ SIRT1 regulates the gluconeogenesis/glycolysis pathway by executing fasting signals via PGC-1α. Augmenting NAD^+^ induces SIRT1-mediated deacetylation of PGC-1α, thereby increasing the production of lipolysis product β-hydroxybutyrate and the production of PGE2, a prostaglandin to maintain kidney function.^593,599^ Additionally, supplementation with NMN restores the activity of kidney SIRT1, thereby reducing the stress response by regulating the JNK pathway and protecting mice from cisplatin-induced AKI.^588^

## Boosting NAD^+^ as a therapeutic strategy

In general, intracellular NAD^+^ levels are maintained between 0.2 and 0.5 mM, depending on the cell type or tissue. However, the concentration and distribution of NAD^+^ can fluctuate in response to diverse physiological stimuli and cellular stresses. Altered NAD^+^ homeostasis has been linked to multiple diseases affecting different organs, including the brain and nervous system, liver, heart and kidney. NAD^+^ depletion is a hallmark of ageing and numerous age-related disorders.^237,238,599,602–605^ Therefore, boosting NAD^+^ offers a promising option for enhancing resilient to aging or diseases, thereby extending a healthy lifespan.^23^ The NAD^+^ level can be elevated by dietary supplementation of NAD^+^ precursors, such as Trp, NA, NMN and NR, inhibition of NAD^+^-consuming enzymes, including PARP1 and CD38, management of the NAD^+^ biosynthesis via controlling NAD^+^-biosynthesis enzymes, or improving NAD^+^ bioavailability through exercise and caloric restriction.

### Supplementation of NAD^+^ precursors

NAD^+^ precursors can be used as a nutritional supplement to improve a broad spectrum of physiological functions and pathological processes.^606–611^ As highlighted in Table 1, the therapeutic and preventive efficacy of NAD^+^ boosters, especially the soluble and orally bioavailable endogenous molecules NR, NAM and Niacin, have been assessed in a series of clinical trials in humans.

**Table 1:** Therapeutic potential of NAD^+^ boosters in human

Human diseases in ICD-11 classification	Conditions	Interventions	NCT Number
Diseases of the circulatory system	Acute coronary syndrome	Niacin	NCT00855257
	Aortocoronary saphenous vein Bypass graft atherosclerosis	Niacin	NCT01221402
	Arterial occlusive diseases	Niacin	NCT00000539
	Atherogenic dyslipidemia	Niacin	NCT03615534
	Atherosclerosis	Niacin; NR	NCT00150722; NCT00127218; NCT02812238
	Cardiovascular diseases	Niacin	NCT02322203; NCT00461630; NCT01200160; NCT00000512; NCT00000483; NCT00000482; NCT00000553; NCT00000461; NCT00880178; NCT00246376; NCT01921010
	Carotid atherosclerosis	Niacin	NCT00804843
	Heart failure	Niacin; NR	NCT00458055; NCT00590629; NCT02003638; NCT01178320; NCT00715273; NCT00298909; NCT01126073; NCT03423342; NCT03727646
	Established carotid atherosclerosis	Niacin	NCT00307307
	Intermittent claudication	Niacin	NCT00062556; NCT00071266
	Peripheral artery disease	Niacin; NR	NCT00687076; NCT03743636
	Pregnancy induced hypertension	Nicotinamide	NCT02213094
	Vascular diseases	NR	NCT04040959
Diseases of the genitourinary system	Acute kidney injury	Niacinamide; NR	NCT02701127; NCT04342975; NCT03176628
	Chronic kidney disease	Nicotinamide; Niacin; NR	NCT02258074; NCT01200784; NCT00852969; NCT03579693
	Polycystic kidney disease	Niacinamide	NCT02558595; NCT02140814
Mental, behavioral or neurodevelopmental disorders	Acute schizophrenia	Niacinamide	NCT00140166
	Depressive disorder	Niacin	NCT03866174
	Obsessive-compulsive disorder	Niacin	NCT03356483
	Post traumatic stress disorder	Niacin	NCT03752918
	Schizophrenia	Niacin	NCT02458924
Aging	Aging	NR; Niacin and Nicotinamide	NCT02921659; NCT03821623; NCT03310034
Diseases of the nervous system	Alzheimer’s disease	Nicotinamide	NCT00580931; NCT03061474
	Chemotherapy-induced peripheral neuropathy	NR	NCT04112641; NCT03642990
	Cognitive function	NR	NCT03562468
	Diabetic neuropathy peripheral	NR	NCT03685253
	Ischemic stroke	Niacin	NCT00796887
	Mild cognitive impairment	NR	NCT03482167; NCT02942888
	Multiple sclerosis	Niacinamide	NCT01381354
	Parkinson’s disease	Niacin; NR	NCT03808961; NCT03462680; NCT03816020
	Progressive supranuclear palsy	Niacinamide	NCT00605930
	Retinal vein occlusion	Niacin	NCT00493064
Others	Bioavailability	NR	NCT02712593
	Cystic fibrosis	NR	NCT04166396
	Development	Nicotinamide	NCT03268902
	Flushing	Niacin	NCT00930839; NCT00533611; NCT00536237
	Gulf War illness	Niacin	NCT01672710
	Healthy	Nicotinamide; Niacin; NR	NCT03136705; NCT03974685; NCT01809301; NCT00359281; NCT01258491; NCT00608699; NCT01275300; NCT00953667; NCT03838822; NCT02191462; NCT02678611; NCT03151707; NCT02300740; NCT03818802
	Melasma	Niacinamide	NCT03392623
	Muscle injury	NR	NCT03754842
	Preeclampsia	Nicotinamide	NCT03419364
	Psychosis	Niacin	NCT01720095
	Recovery of function	NR	NCT03635411
	Sickle cell disease	Niacin	NCT00508989
	Sleep apnea	Niacin	NCT04234217
	Inflammation	NR	NCT04110028
Neoplasms	Bladder cancer	Niacinamide	NCT00033436
	Non-melanoma skin cancer	Niacinamide	NCT03769285
	Non-small-cell lung carcinoma	Niacinamide	NCT02416739
	Non-Hodgkin’s lymphoma	Niacin; Niacinamide	NCT00957359; NCT02702492; NCT04281420; NCT00691210
	Cancer	NR	NCT03789175
	Multiple myeloma	Nicotinamide	NCT03019666
Diseases of the skin	Contact dermatitis of hands	Niacinamide	NCT04218500
	Hand-foot skin reaction	Niacin	NCT04242927
	Hyperpigmentation	Niacinamide	NCT01542138
	Psoriasis	Nicotinamide; NR	NCT01763424; NCT04271735
Endocrine, nutritional or metabolic diseases	Diabetes mellitus type 2	Niacin	NCT00485758; NCT03685773; NCT00618995; NCT02153879; NCT03867500
	Dyslipidemia	Niacin	NCT00903617; NCT00194402; NCT01984073; NCT00944645; NCT00943124; NCT00626392; NCT00111891; NCT00111891; NCT00728910; NCT00961636; NCT01803594; NCT02642159; NCT01104519; NCT01071291; NCT00079638; NCT01250990
	Glucose metabolism disorders	NMN supplement	NCT03151239
	Hypercholesterolemia	Niacin	NCT00376584; NCT02890992; NCT03510884; NCT01321034; NCT00769132; NCT00080275; NCT00082251; NCT00271817; NCT00533312; NCT01054508; NCT03510715; NCT00378833; NCT00652431; NCT00536510
	Hyperlipidemia	Niacin	NCT00244231; NCT00203476; NCT00465088; NCT00345657
	Hyperphosphatemia	Niacinamide	NCT00508885; NCT00316472
	Insulin sensitivity	Niacin	NCT01216956
	Metabolic disturbance	NR	NCT02689882
	Metabolic syndrome	Niacin	NCT00300365; NCT00346970; NCT02061267; NCT00286234; NCT00304993
	Mitochondrial diseases	Niacin,NR	NCT03973203; NCT03432871
	Obesity	Niacin,NR	NCT01083329; NCT02303483; NCT03951285; NCT02835664
	Polycystic ovary syndrome	Niacin	NCT01118598
	Primary hypercholesterolemia	Niacin	NCT00269204; NCT00479388; NCT01012219; NCT00269217
	Undernutrition	Nicotinamide	NCT04012177
Infection	HIV infections	Niacin,NR	NCT02018965; NCT00986986; NCT00046267; NCT01426438; NCT00202228
Diseases of the digestive system	Non alcoholic fatty liver disease	Niacinamide	NCT03850886; NCT00262964; NCT04330326
Diseases of the blood or blood-forming organs	Sickle cell disease	Nicotinamide	NCT04055818

#### NAD^*+*^ precursor: NMN

NMN administration can effectively and rapidly enhance NAD^+^ biosynthesis in various tissues, even in the brain, with a promising safety.^612^ Aged animals are more responsive to NAD^+^ replenishment by NMN treatment than the young one due to the age-related decline in NAD^+^ availability. NMN treatment exerts beneficial effects on insulin secretion and insulin sensitivity in age- and diet-induced diabetes by restoring NAD^+^ biosynthesis. Long-term NMN administration rescues the age-associated decline in physiological function, including mitochondrial function, energy metabolism, gene expression changes, insulin sensitivity and plasma lipid profile, thereby improving physical activities, such as bone density and eye function.^612^ NMN treatment improves the neuronal functions, including rescuing the memory and cognition in rodent models for Alzheimer’s disease, protecting neurons from cell death after intracerebral hemorrhage or ischemia, recovering severe retinal degeneration and restoring the age-associated loss of the neural stem/progenitor pool. Besides, the NMN administration also exerts a pleiotropic effect on acute heart failure and renal injury.^23,613,614^ It is worth noting that clinical trials of NAM have been initiated in patients with cancers including bladder cancer, non-small-cell lung carcinoma, non-melanoma skin cancer, non-Hodgkin’s lymphoma and multiple myeloma (Table 1).

Despite that the NMN bioavailability is evidenced by the rapid absorption and convention of administered NMN to NAD^+^ in various organs, including skeletal muscle, kidney and liver, the transportation of NMN into cells remains unclear. NMN may be directly taken up by specific transporters, as the NAD^+^ content in peripheral organs, such as the gut, is immediately increased by NMN administration.^23,614^ However, in vitro studies demonstrate that Nrk1/2 depletion abandons the incorporation of NMN into NAD^+^ synthesis. Moreover, NMN administration can significantly elevate NAD^+^ biosynthesis in white adipose tissue, heart and the liver, but not the NAD^+^ content in brown adipose tissue and kidney, suggesting a tissue- and cell-type-specific transportation of NMN to cells or tissues for NAD^+^ biosynthesis presumably due to the deferent expression pattern of NRK1.^612,615^ Therefore, the identification of the putative NMN transporter and its tissue specific expression pattern will help assess the cell type or tissue-specific preference of NMN, paving the way for precious clinical application of NMN in different conditions.

#### NAD^+^ precursor: NR

NR is another natural compound that displays a surprisingly robust effect on systemic NAD^+^ metabolism. A large phase clinical trial of NR has been registered on a broad range of pathologies, including infection, neoplasms, aging-related diseases and disorders that occur in the circulatory system, genitourinary system, nervous system and skin (Table 1). Oral NR supplementation in aged participants elevates the muscle NAD^+^ metabolome, ameliorates metabolic dysfunction, depresses levels of circulating inflammatory cytokines and increases the anti-inflammatory molecule adiponectin in aged human.^606,616,617^ Dietary administration with NR improves cold tolerance, endurance and energy expenditure. NR protects mice from HFD-induced body weight gain, enhances the liver weight regain by promoting hepatocyte replication and increasing hepatic ATP content in the regenerating liver.^618^ NR exhibits beneficial effects in several muscle disorders through improving mitochondrial function and decreasing the UPR^mt^ in heart failure mice. NR boosts the NAD^+^ biosynthesis to prevent DNA damage and tumorigenesis. NAD^+^ repletion with NR may reverse NAFLD by improving mitochondrial function in both HFHS-fed mice and HFC‐fed Apoe^−/−^ mice.^619^ It has been reported that NR has a variety of compelling benefits in the nervous system, including improving the cognitive function and synaptic plasticity in Alzheimer’s disease and preventing noise-induced hearing loss. NR restores the age-associated decline in the metabolic cycle and circadian behavioral, including the BMAL1 activity, oscillation mitochondrial respiration, rhythmic transcription and late evening activity to youthful levels.^242^

NR can be directly transported by ENTs into cells and enhance NAD^+^ biosynthesis bypassing the NAMPT-mediated salvage pathway. However, the short stability of NR in circulation and rate-limited utilization by the expression of NRKs restrict its clinical application. Dihydronicotinamide riboside (NRH), a reduced form of NR with oral bioavailability, is developed to overcome these limitations. NRH provides better efficacy to boost NAD^+^ synthesis using an NRK1/2-independent pathway compared with NR and NMN, preventing cisplatin-induced acute kidney injury. The potent and efficient NRH that serve as an NAD^+^ booster, offers a promising option to increase NAD^+^ levels.^620,621^

#### NAD^+^ precursors: NAM and NA

NAM is an uncharged molecule that can diffuse rapidly across the plasma, supporting the NAD^+^ biosynthesis for most tissues in vivo.^91^ Oral administrated NAM is converted into NA in the small intestine and colon by nicotinamidase PncA of gut microbiota. Gut microbiota-mediated deamidation of NAM is necessary and responsible for the NAD^+^ biosynthesis in various organs, including the kidney, the liver and the colon.^101^ NAM protects against streptozotocin (STZ)-induced diabetes by recovering the NAD^+^ decline in pancreatic islet cells. NAM treatment exhibits profound metabolic improvements in obesity and type 2 diabetes mouse model. However, there are several side effects caused by NAM, limiting the application of NAM. Firstly, NAM exerts feedback inhibition on SIRT1 activity. Secondly, it has been indicated that high doses or long-term NAM reduces the methyl group availability and cellular methylation potential via promoting the methyl sink in the form of 1‐MNA. Consistent with this hypothesis, dietary methionine supplementation attenuates the development of steatohepatitis induced by high doses of NAM.^66,622^

NA is effective in treating dyslipidemia due to its cholesterol lowering actions. NA treatment decreases the serum low-density lipoprotein and triglyceride content and elevates the high-density lipoprotein levels. Nevertheless, the clinical application of NA is limited because the pharmacological dosing of it induces cutaneous flushing via activation of a G protein-coupled receptor, GPR109A. Given this undesirable effect, several niacin derivatives with prolonged release time, including enduracin, niaspan and acipimox, have been developed. Therefore, niacin has been replaced by its derivatives in the clinical treatment of hyperlipidemia. The Acipimox can directly affect mitochondrial function in skeletal muscle of patients with type 2 diabetes.^623^

#### The side effects of NAD^+^ boosting

The aforementioned findings suggest that elevating NAD^+^ levels by administration of NAD^+^ precursors, including NMN, NR, NAM, and NA, is a rational therapeutic strategy to improve a healthy lifespan. Given that NAD^+^-depleting drugs exhibit anti-tumor potential due to their impact on DNA repair and inflammation, long-term boosting NAD^+^ might increase the risk of driving tumor growth. Moreover, the detrimental side effects of NAD^+^ and its intermediates may be caused by the NAD^+^-dependent sirtuins that have both oncogenic and tumor suppressive activity in different contexts. Consistent with this hypothesis, NMN treatment accelerates pancreatic cancer progression via creating an inflammatory environment.^66,260^ Thus, future clinical studies are necessary to assess the long-term safety of NAD^+^ precursors in human therapeutics.

### Inhibition of NAD^+^ consumption

The excessive activation of PARPs or CD38 causes a NAD^+^ consumption up to the extent that leads to ATP decline, energy loss and cell death.^52,184,624,625^ Thus, reducing the NAD^+^ consumption via suppressing PARPs or CD38 is also a strategy to boost NAD^+^.^53,626^

Accumulating evidence demonstrates that aberrant PARPs activation by DNA damage causes NAD^+^ depletion, contributing to the progression of tumorigenesis and neurodegenerative disorders that involve DNA repair defects. To date, PARP inhibitors, including niraparib, rucaparib and olaparib have been approved by US-FDA to treat cancers, including prostate cancer, breast cancer and ovarian cancer, through disrupting DNA repair and replication pathways.^627–629^ PARPs-mediated ADP-ribosylation accounts for up to 90% of the cellular intracellular NAD^+^ consumption, leading to reduced NAD^+^ availability for sirtuins. Therefore, genetic ablation or pharmacological inhibition of Parp-1 enhances the Sirt1 activity through restoring the NAD^+^ content, providing a protection benefit for various tissues, including the liver, muscle and brown adipose tissue.^630,631^ NADP^+^ has been demonstrated as an endogenous inhibitor of PARPs, which extend the therapeutic effect of PARP inhibitors on cancers with higher levels of NADP^+^.^184^

A variety of flavonoids, including apigenin, quercetin, luteolin, kuromanin, and luteolinidin, exhibit inhibitory effect on CD38 activity.^23^ 78c is a highly specific CD38 inhibitor, which has greater potency than the flavonoids in reversing NAD^+^ decline during aging, thereby improving several age-associated physiological functions, including cardiac function, muscle function and glucose tolerance.^53^ Interestingly, 78c elevates NAD^+^ to a higher level in mouse liver than that in muscle, arguing a tissue specific CD38 activity.^632^ Thus, further studies uncovering the tissue specific CD38 activities will facilitate the development of clinical application of CD38 inhibitors.

### Controlling NAD^+^-biosynthesis pathway

Enhancing NAD^+^-biosynthesis is one alternative approach to elevate NAD^+^ concentration through either increasing the activity of enzymes in NAD^+^-biosynthetic pathway or inhibiting the activity of enzymes in the side branch pathway.^633^

NAD^+^ is synthesized from both de novo pathway and the salvage pathway.^634^ The distinct expression level of NAPRT in various healthy tissues determines the choice of NAD^+^ biosynthetic pathway for survival. Cancers arising from tissue with a highly NAPRT are expression completely and irreversibly dependent on the NAPRT-regulated de novo pathway, while cancers deriving from tissues with the low level of NAPRT mainly rely on the NAMPT-mediated NAD^+^ salvage pathway. This deferent dependence renders cancer cells resistant to inhibition of NAMPT by other NAD^+^ synthesis.^408^ In line with this hypothesis, the loss of NAPRT in both RCC cell lines and EMT-subtype gastric cell lines renders the cells hypersensitive to NAMPT inhibitors, such as FK866, and KPT-9274, suggesting that NAMPT inhibitors may be a promising strategy for NAPRT deficient tumors.^421,423^ Moreover, bacteria-mediated deamidated NAD^+^ biosynthesis also rescues NAMPTi-induced toxicity in cancer cells and xenograft tumors.^101^

The enzymatic ability of NAMPT can be enhanced by pharmacological agents, P7C3 or SBI-797812. P7C3 is a NAMPT enhancer with good bioavailability and nontoxicity. It has been demonstrated that P7C3 and its analogs have neuroprotective efficacy in a broad range of preclinical rodent and nonhuman primate models relying on the activation of NAMPT.^635–638^ Therefore, the neuroprotective activity of P7C3 offers a new pharmacotherapy for age-associated ALS, Alzheimer’s disease and Parkinson’s disease.^635,637,638^ SBI-797812 activates NAMPT via stabilizing the NAMPT phosphorylation at His247, enhancing the efficiency of NMN generation, providing another option to raise NAD^+^.^639^ Together, the NAMPT enhancers, P7C3 and SBI-797812, warrant further study for the clinical treatment of neuron related diseases.

The NAD^+^-biosynthetic pathway can also be increased by blocking the side branch to prevent the escape of intermediates. Overexpressing ACMSD reduces the NAD^+^ level by dissipating ACMS from the de novo NAD^+^ synthesis into the side branch pathway for acetyl-CoA production, while inhibiting ACMSD elevates NAD^+^ concentration. The high expression of ACMSD in the kidney and liver offers the therapeutic potential of ACMSD inhibitors, such as TES-991 and TES-1025, for renal and hepatic dysfunction.^15,640^ ACMSD may also be a novel target for Parkinson’s disease, as it inhibits the generation of neurotoxin quinolinic acid in the kynurenine pathway.^641^ Similarly, NNMT shifts the NAM into producing 1-methylnicotinamide, leading to impaired salvage pathway. WAT- and liver-specific knockdown of NNMT prevent diet-induced obesity through enhancing the energy expenditure. The effect of NNMT is achieved by its effect on histone methylation. Pharmacological inhibition of NNMT significantly shows benefits diet-induced obese mice, including reducing the body weight gain and adipocyte size, and decreasing serum cholesterol levels.^642,643^ These results suggest that NNMT is an appealing target for obesity and type 2 diabetes treatment.^642^ Together, ACMSD and NNMT provide novel targets for modulating NAD^+^ homeostasis, which will be of great importance to determine whether ACMSD and NNMT inhibitors can increase NAD^+^ levels and achieve therapeutic effects.

### Increasing NAD^+^ bioavailability

Intracellular NAD^+^ levels can also be increased by energy stress, including fasting, glucose restriction, caloric restriction (CR) and exercise.^599^ CR-mediated NAD^+^ boosting depends on the NAD^+^ salvage biosynthesis rather than de novo pathway via elevating the NAMPT expression.^237,238,644–646^ CR restores the age-associated circadian decline via sharpening circadian control of NAD^+^ metabolism and NAD^+^/SIRT1-modulated epigenetic modification.^647–650^ It has been shown that both long-term and short-term CR rescue the large elastic artery stiffening and endothelial dysfunction.^651,652^ Similarly, CR-boosted NAD^+^ level protects the brain against aging and diseases through attenuating plasma membrane lipid peroxidation, protein carbonyls, nitrotyrosine and oxidative stress.^653^

Beyond CR, the exercise has attracted growing attention due to its benefits on health. In this context, exercise could also increase the NAD^+^ level and SIRT1 activity due to an increase in NAMPT.^605^ It has been reported that the NAMPT protein levels in athletes’ skeletal muscle is much higher than in type 2 diabetic, sedentary obese and nonobese subjects. Furthermore, exercise training induces a robust increase in the skeletal muscle of NAMPT protein in sedentary nonobese subjects.^605^ The exercise-mediated NAD^+^ boosting by inducing *Nampt* is a response strategy for energy stress, which is abolished by depletion of the energy sensor AMPK.^603^ Intriguingly, the gut microenvironment, especially the host-bacteria interactions also contributes to NAD^+^ metabolism. The gut microbiota-derived deamidated pathway facilitates the utilization of NAM or NR for hepatic NAD^+^ synthesis, suggesting that manipulation of microbiota might offer a new option to manipulate NAD^+^ metabolism.^101^ Taken together, both caloric restriction and exercise provide the potential therapeutic strategies in therapy against pathologies related to NAD^+^ decline.

## Concluding remarks

The levels and compartmentalization of NAD^+^ dictate energy state that impinges on normal physiological and biological responses, as indicated by the regulatory role of NAD^+^ in proper redox homeostasis, genomic stability, gene expression, circadian clock, inflammation, metabolism, cellular bioenergetics, mitochondrial homeostasis and adaptive stress responses. A healthy lifestyle and exercise are non-pharmacologic strategies to improve the body’s resilience and extend healthy lifespan through enhancing NAD^+^ levels. NAD^+^ boosters can be applied for a broad spectrum of NAD^+^ deficiency related pathologies, such as infection, cancer, metabolic diseases, acute injury, aging and aging-related neurodegenerative disorders. Conceivably, this could be achieved by boosting NAD^+^ via enhancing the NAD^+^ generation and diminishing NAD^+^ consumption.

Despite exciting and emerging strides in NAD^+^ biology, there are a variety of outstanding questions that warrant future systematic exploitation to accelerate the translation of remarkable bench work to effective clinical application in humans. The first interesting question is that the precise mechanisms executing the beneficial effects of NAD^+^ and its metabolites on pathologies and lifespan remain elusive. Further investigation understanding the landscape of NAD^+^ in response to diseases and identifying the specific effector molecules for each NAD^+^ precursors at different time points provide critical insights into development of effective interventions for various physiologies. Secondly, the systemic NAD^+^ metabolome is largely unexplored. Are there any tissue specificities for NAD^+^ boosting, such tissue preferences of distinct NAD^+^ precursors? What is the crosstalk with the NAD^+^ systems of each organ? What is the distinct NAD^+^ metabolome in each tissue? In spite of growing interest in the use of NAD^+^ precursors as a strategy for healthy aging, the in vivo pharmacokinetics remain poorly understood. The efficacy of NAD^+^ boosters, the therapeutic dosages and favorable administration routes should be optimized for different diseases in humans. It is also essential to fully assess the unforeseen side effects of long-term NAD^+^ boosting. This task requires the development of new technologies to enable the simplifying, accurate and reproducible monitoring of dynamic NAD^+^ and its metabolites in patients and healthy individuals.
